# Genome-wide CRISPR/Cas9 screening identifies a targetable MEST-PURA interaction in cancer metastasis

**DOI:** 10.1016/j.ebiom.2023.104587

**Published:** 2023-05-05

**Authors:** Wen Wen Xu, Long Liao, Wei Dai, Can-Can Zheng, Xiang-Peng Tan, Yan He, Qi-Hua Zhang, Zhi-Hao Huang, Wen-You Chen, Yan-Ru Qin, Kui-Sheng Chen, Ming-Liang He, Simon Law, Maria Li Lung, Qing-Yu He, Bin Li

**Affiliations:** a Key Laboratory of Biological Targeting Diagnosis, Therapy and Rehabilitation of Guangdong Higher Education Institutes and Key Laboratory of Protein Modification and Degradation, The Fifth Affiliated Hospital of Guangzhou Medical University and School of Basic Medical Sciences, Guangzhou Medical University, Guangzhou, China; b MOE Key Laboratory of Tumor Molecular Biology, National Engineering Research Center of Genetic Medicine, College of Life Science and Technology, Jinan University, Guangzhou, China; c Department of Clinical Oncology, Li Ka Shing Faculty of Medicine, The University of Hong Kong, Hong Kong, China; d Research Center of Cancer Diagnosis and Therapy, and Department of Clinical Oncology, First Affiliated Hospital, Jinan University, Guangzhou, China; e Department of Thoracic Surgery, First Affiliated Hospital, Jinan University, Guangzhou, China; f State Key Laboratory of Esophageal Cancer Prevention and Treatment, Department of Clinical Oncology, First Affiliated Hospital, Zhengzhou University, Zhengzhou, China; g Henan Province Key Laboratory of Tumor Pathology, Department of Pathology, First Affiliated Hospital, Zhengzhou University, Zhengzhou, China; h Department of Biomedical Sciences, City University of Hong Kong, Hong Kong, China; i Department of Surgery, Li Ka Shing Faculty of Medicine, The University of Hong Kong, Hong Kong, China

**Keywords:** Cancer metastasis, CRISPR/Cas9 screening, Protein interactome, Noncoding RNA, Esophageal cancer

## Abstract

**Background:**

Metastasis is one of the most lethal hallmarks of esophageal squamous cell carcinoma (ESCC), yet the mechanisms remain unclear due to a lack of reliable experimental models and systematic identification of key drivers. There is urgent need to develop useful therapies for this lethal disease.

**Methods:**

A genome-wide CRISPR/Cas9 screening, in combination with gene profiling of highly invasive and metastatic ESCC sublines, as well as PDX models, was performed to identify key regulators of cancer metastasis. The Gain- and loss-of-function experiments were taken to examine gene function. Protein interactome, RNA-seq, and whole genome methylation sequencing were used to investigate gene regulation and molecular mechanisms. Clinical significance was analyzed in tumor tissue microarray and TCGA databases. Homology modeling, modified ELISA, surface plasmon resonance and functional assays were performed to identify lead compound which targets MEST to suppress cancer metastasis.

**Findings:**

High MEST expression was associated with poor patient survival and promoted cancer invasion and metastasis in ESCC. Mechanistically, MEST activates SRCIN1/RASAL1-ERK-snail signaling by interacting with PURA. miR-449a was identified as a direct regulator of MEST, and hypermethylation of its promoter led to MEST upregulation, whereas systemically delivered miR-449a mimic could suppress tumor metastasis without overt toxicity. Furthermore, molecular docking and computational screening in a small-molecule library of 1,500,000 compounds and functional assays showed that G699-0288 targets the MEST-PURA interaction and significantly inhibits cancer metastasis.

**Interpretation:**

We identified the MEST-PURA-SRCIN1/RASAL1-ERK-snail signaling cascade as an important mechanism underlying cancer metastasis. Blockade of MEST-PURA interaction has therapeutic potential in management of cancer metastasis.

**Funding:**

: This work was supported by 10.13039/501100012166National Key Research and Development Program of China (2021YFC2501000, 2021YFC2501900, 2017YFA0505100); 10.13039/501100001809National Natural Science Foundation of China (31961160727, 82073196, 81973339, 81803551); NSFC/RGC Joint Research Scheme (N_HKU727/19); 10.13039/501100003453Natural Science Foundation of Guangdong Province (2021A1515011158, 2021A0505030035); Key Laboratory of Guangdong Higher Education Institutes of China (2021KSYS009).

**Research in context**

**Evidence before this study**

The underlying mechanisms remain unclear in ESCC metastasis, which represents a main obstacle to cancer treatment. A comprehensive investigation of key drivers in regulating cancer metastasis is urgently needed.

**Added value of this study**

Integrative analysis of CRISPR/Cas9 screening and gene profiling of metastatic cell/animal model identifies MEST as a driver of cancer metastasis. MEST activates SRCIN1/RASAL1-ERK-snail signaling by interacting with PURA, which could be targeted by compound G699-0288.

**Implications of all the available evidence**

Our study validates MEST as a promising prognostic biomarker and therapeutic target for ESCC patients. Blockade of MEST-PURA interaction has therapeutic potential in the management of cancer metastasis.

## Introduction

Metastasis accounts for approximately 90% of cancer-related deaths.^1^ As a complex process, metastasis consists of a series of sequential steps, including cancer cell invasion, survival in blood vessels, extravasation and colonization in the distant organs.^2^ Each of these steps is driven by the acquisition of genetic and/or epigenetic alterations within the tumor. Although cell subpopulations with heterogeneous metastatic potential that pre-exist in the tumor have been recognized as a major obstacle to treatment,^3^ metastasis remains the least understood aspect of cancer, and in particular, there is little information and functional validation of metastasis-associated genes in esophageal squamous cell carcinoma (ESCC). Therefore, a comprehensive investigation of key drivers and signaling pathways in regulating cancer metastasis is urgently needed, which would provide new clues for cancer therapy.

Genome-wide clustered regularly interspaced short palindromic repeats (CRISPR)/Cas9 screening is an ideal tool for systematic identification of key genes involved in specific biological processes.^4^ In addition, the study of cancer metastasis has been hampered by a lack of cell and animal models. In this study, on one hand, we performed a functional screening with a genome-wide CRISPR/Cas9 library to identify the key drivers in cancer metastasis; one the other hand, we established highly invasive and metastatic sublines by serial *in vitro* and *in vivo* selection of cancer cells that were able to invade through Matrigel-coated Boyden chambers and metastasized to the lungs of mice, respectively.^5^^,^^6^ Among the overlapped genes between the two databases obtained, we have validated mesoderm-specific transcript (MEST) as a critical promoter of ESCC metastasis. MEST, located at the chromosome 7q32 region, is also known as paternally expressed gene 1 (PEG1) and is highly expressed in the mesodermal layer. Up to now, the biological function of MEST in cancer is unknown.

The present study provides evidence suggesting that overexpression of MEST is a common event in ESCC, and it highlights the role of MEST as a potential driver of cancer metastasis. Deciphering the function of MEST in tumor invasion and metastasis has great functional significance, and elucidating the upstream and downstream mechanisms of MEST will provide mechanistic insight into cancer progression. We also aimed to discover small molecules that can inhibit the MEST-mediated signaling pathway and suppress tumor metastasis *in vitro* and *in vivo*.

## Methods

### Cell lines and drugs

The human ESCC cell lines KYSE150 (CVCL_1348), KYSE270 (CVCL_1350), KYSE30 (CVCL_1351) and KYSE410 (CVCL_1352) obtained from DSMZ (Braunschweig, Germany). The EC9706 esophageal cancer cell line (CVCL_E307) was purchased from the Cell Bank of Type Culture Collection of the Chinese Academy of Sciences (Shanghai, China).^7^^,^^8^ These cell lines were maintained in RPMI 1640 (Thermo Fisher Scientific, Waltham, MA, USA) supplemented with 10% fetal bovine serum (FBS) (Invitrogen, Gaithersburg, MD, USA). The immortalized normal esophageal epithelial cell line SHEE was obtained from Shantou University,^9^^,^^10^ and the NE1, NE3 and NE6 cell lines were gifts from Prof. George Tsao and Dr. Annie Cheung,^11^, ^12^, ^13^ and these cell lines were maintained in DMEM (Thermo Fisher Scientific) supplemented with 10% FBS. Luciferase-expressing KYSE150-Luc and EC9706-Luc cell lines were generated as previously described.^5^ All cell lines used were cultured within 35 generations and tested negative for mycoplasma throughout the study. The short tandem repeat profiling was used to verify the identity of cell lines. KYSE150-Luc-LM5 were generated via serial injection and selection in mice. In brief, KYSE150-Luc cells were intravenously injected into the mice through the tail vein. After 30 days, the lung tissue with metastases was isolated and chopped to pieces. Luciferase-positive cells were selected by blasticidin and named as KYSE150-Luc-LM1. The above steps were repeated for 5 rounds to obtain KYSE150-Luc-LM5. U0126 was purchased from Cell Signaling Technology (Beverly, MA, USA), and 5-aza-2′-deoxycytidine (5-Aza) was obtained from Sigma–Aldrich (St. Louis, MO, USA).

### Genome-wide CRISPR/Cas9 screening

The GeCKO v2 human library (#1000000048) was purchased from Addgene and prepared as described previously.^14^ For the genome-wide screening, cells were transduced at a multiplicity of infection (MOI) of approximately 0.3 to obtain coverage of at least 500-fold per gRNA. In brief, the GeCKO v2 human library was transfected into 293T cells, the virus-containing culture supernatant was collected to infect ESCC cells KYSE150-Luc. ESCC cells were treated with puromycin 48 h after transduction and 2 × 10^7^ cells were harvested one week later to obtain input DNA. Remaining cells were selected via Matrigel-coated Boyden chambers. The cells that invaded and adhered to the lower surface of the chamber were detached with trypsin and cultured until the number of cells was adequate for the next round of invasion selection, Then, the cells were reseeded into the upper compartment of a new invasion chamber. The same procedure was repeated three times. At least 7 × 10^7^ cells were maintained at any given time to ensure sgRNA representation. After the third round of screening, cells were harvested and genomic DNA extracted using a Blood & Cell Culture DNA Midi Kit (Qiagen, Hilden, Germany). sgRNA inserts were PCR-amplified from 10 μg gDNA using NEBNext High Fidelity PCR Master Mix (NEB, Ipswich, MA, USA). PCR products were used to construct paired-end libraries using Paired-End DNA Sample Prep kit (Illumina Inc., San Diego, CA, USA). Deep sequencing was performed using novaseq6000 (Illumina Inc., San Diego, CA, USA) NGS platform at Genedenovo company (Guangzhou, China). Reads obtained from the sequencing machines includes raw reads containing adapters or low-quality bases which will affect the following assembly and analysis. Thus, raw reads would be processed to get high quality clean reads according to following stringent filtering standards: 1) removing reads with ≥10% unidentified nucleotides (N); 2) removing reads with >40% bases having phred quality scores of ≤20; 3) removing reads aligned to the barcode adapter. Blastn (version2.6.0+; parameter: -word_size 18) was used for mapping reads to sgRNA library. For all sgRNA, based on their expression in each sample, the sgRNA expression level was calculated and normalized to transcripts per million (TPM). The formula is as follows: TPM=Actual miRNA counts/Total counts of clean tags∗10^6^. To identify differentially expressed sgRNAs across samples or groups, Kolmogorov–Smirnov (ks) test analysis was performed by R language. The sgRNAs with fold change <0.5 and *P* value < 0.005 in a comparison were deemed as significantly depleted sgRNAs.

### Plasmids, transfection, and infection

The plasmids expressing MEST, PURA and the vector control, as well as the shRNAs against MEST (shMEST), PURA (shPURA) and the scrambled negative control (shCON), were obtained from TranSheepBio (Shanghai, China). To generate MEST overexpression stable cell lines, plasmid containing the MEST cDNA was co-transfected with the 3rd generation packaging plasmids (Addgene_12251, Addgene_12253, Addgene_12259) into HEK293T cells (CVCL_0063) using Lipofectamine 3000 as previously described.^5^^,^^15^ Supernatants were collected 48 h after transfection, centrifuged at 1500 rpm for 5 min, and filtered through a 0.45 μm filter. ESCC cells were infected to produce stable cell lines. After 7 days of puromycin selection (1 μg/ml), the MEST protein level was analyzed by Western blot. The BLOCK-iT™ Pol II miR RNAi Expression Vector Kit with EmGFP (Invitrogen, #K493600) was used to construct the vectors expressing miR-449a and the scrambled miRNA control (miR-CON). The miR-449a mimic (#4464066) and negative control (#4464058) were ordered from Thermo Fisher Scientific. The miRIDIAN anti-miR-449a inhibitor (IH-300723-05) and the corresponding negative control (IN-001005-01) were purchased from GE Healthcare Dharmacon (Lafayette, CO, USA).

### CRISPR/Cas9-mediated genome editing

CRISPR/Cas9-mediated genome editing was performed as previously described.^16^ In brief, the pLentiCRISPR/Cas9 V2 vector (Addgene_52961) expressing the sgRNA targeting PURA and MEST was transfected into 293T cells. After infection, KYSE150-Luc-PURA-KO and KYSE150-Luc-MEST-KO cells were selected using puromycin. Successful knockout of the targeted gene was validated by Western blotting and genomic DNA sequencing.

### ESCC samples and tissue microarray

Fresh human ESCC tumor samples and the corresponding adjacent nontumorous esophageal samples were collected with informed consent from 40 patients who were treated with surgical resection without receiving neoadjuvant chemoradiotherapy at First Affiliated Hospital, Zhengzhou University, Zhengzhou. The use of all human samples was approved by the committee for ethical review of research involving human subjects at Zhengzhou University (SS-2020-003). Two tissue microarrays consisting of 242 ESCC and 212 corresponding normal tissues were obtained from Shanghai Outdo Biotech Co., Ltd. (Shanghai, China) and another tissue microarray containing 40 pairs of primary ESCC and metastatic tissues were obtained from Biomax (Rockville, MD, USA). The use of the tissue microarrays was ethically approved (SHYJS-CP-1804005, SHYJS-CP-1501003).

### Immunohistochemical staining

Immunohistochemical staining was performed by investigators blinded to sample identity as previously described.^5^ The MEST primary antibody was purchased from Novus Biologicals (Littleton, CO, USA, AB_11006156), and antibodies against SRCIN1(AB_2881315) and RASAL1 (AB_2807382) were obtained from Proteintech (Chicago, IL, USA) and Invitrogen, respectively. Briefly, the tissue microarrays were deparaffinized and rehydrated and then heated for 15 min in 10 mM citrate (pH 6). After incubation with primary antibody at 4 °C overnight followed by corresponding biotinylated secondary antibody, the immunostaining was visualized using peroxidase-conjugated avidin-biotin complex and 3,3′-diaminobenzidine (Dako, Mississauga, ON, Canada) as the chromogen. The sections were counterstained with hematoxylin. The IHC and ISH staining intensity in the TMA was categorized: no staining as 0, weak as 1, moderate as 2, and strong as 3. Spots with 0 or 1 scoring were classified as low expression, while 2 or 3 were high expression.

### *In situ* hybridization assay

The *in situ* hybridization assay was performed as previously described.^17^ The tissue microarray was deparaffinized in xylene and rehydrated with graded alcohol, followed by digestion with 8 mg/ml pepsin. The slides were hybridized with a probe against miR-449a (40 nM) (Exiqon, Vedbaek, Denmark) at 60 °C overnight. The degree of staining was scored similarly to the immunohistochemical staining.

### Whole-genome-based bisulfite sequencing (WGBS)

Genomic DNA was purified with a QIAquick Gel Extraction Kit (Qiagen) and bisulfite treated with a Methylation-Gold Kit (ZYMO, Irvine, CA, USA). The qualified library was amplified on cBot to generate clusters on flow cells (TruSeq PE Cluster Kit V3ocBotth 8 mg/ml pe, San Diego, CA, USA). The flow cells were sequenced for 150 bp of PE reads on the HiSeq X Ten platform, and more than 90 G of clean data was generated (Beijing Genomics Institute Tech). Next, we predicted the methylation sites in miR-449a promoter (http://www.urogene.org/cgi-bin/methprimer/methprimer.cgi).^18^ According to the predicted region, the read coverage of site C in depth ≥4 within the region was calculated. CpG density and GC content of individual hypomethylated regions was calculated based on the underlying DNA sequence for hypomethylated regions.

### Measurement of triglyceride and free fatty acid

Triglyceride assay kit and nonesterified free fatty acids assay kit were used to determine the levels of triglyceride and free fatty acids respectively, according to manufacturer's protocols (Nanjing Jiancheng Bioengineering Institute, Nanjing, China). Briefly, ESCC cells were sonicated in PBS, and incubated at 37° for 10 min after adding the reaction solution. The triglyceride level was quantified by measuring the absorbance of the solution at 510 nm; the free fatty acids level was quantified by measuring the absorbance of the solution at 546 nm.

### Site-directed mutagenesis and luciferase reporter assay

Site-directed mutagenesis and luciferase reporter assays were performed as previously described.^19^ Three software programs, TargetScan (http://www.targetscan.org/vert_50/),^20^ miRanda (http://www.microrna.org/microrna/getExprForm.do)^21^ and PicTar (http://pictar.mdc-berlin.de/cgi-bin/PicTar_vertebrate.cgi),^22^ were used to predict the putative binding sites of miR-449a on the 3′UTR of MEST. The MEST 3′-UTR was cloned into the psiCHECK-2 reporter vector (Promega, Madison, WI, USA, C8021), and mutant variants of the MEST 3′-UTR were created using the QuikChange Lightning Site-Directed Mutagenesis Kit (Agilent Technologies, Santa Clara, CA, USA). For the dual-luciferase reporter assay, the SRCIN1 and RASAL1 promoter regions (named WT) and fragments containing site mutations of the putative binding sites of PURA (named Mut) were cloned into the pGL3.0-Basic vector (Promega, E1751). Luciferase activity was measured by using a Dual-Luciferase Reporter Assay System (Promega) according to the manufacturer's instructions.

### Bisulfite DNA modification and methylation-specific PCR

Genomic DNA was extracted from cells and tissues as previously described.^6^ The extracted DNA was subjected to bisulfite treatment using the Methylation-Gold Kit (ZYMO) according to the manufacturerivity was measurePCR products were analyzed by electrophoresis on a 2% (w/v) agarose gel.

### RNA sequencing (RNA-seq) and ingenuity pathway analysis (IPA)

RNA-seq was performed to compare the gene profiles of MEST-overexpressing cells and parental cells at the Beijing Genomics Institute Tech (Shenzhen, China), and genes with a fold change of >2.0 were defined as differentially expressed. IPA software (Ingenuity Systems, Redwood City, CA, USA) was used for pathway analysis.

### Real-time polymerase chain reaction (RT-PCR) and TaqMan miRNA assay

Total RNA of cells and tissues was isolated using TRIzol reagent (Invitrogen). The mRNA was converted to cDNA with PrimeScript II First Strand cDNA Synthesis Kit (TaKaRa, Dalian, China) according to the manufacturer's protocol, and the mRNA expression of SRCIN1, RASAL1 and GAPDH as the internal control was analyzed using SYBR Premix Ex TaqII (TaKaRa) on a MiniOpticon Real-Time PCR System (Bio-Rad, Hercules, CA, USA). The expression of miR-449a was quantified with the TaqMan miRNA Assay Kit (Applied Biosystems, Carlsbad, CA, USA) according to the manufacturer's instructions. Human small nuclear U6 RNA was included as an internal control for miRNA detection.

### Western blot analysis

The preparation of cell lysates and immunoblotting protocol were similar to those previously described.^5^ The primary antibodies used included antibodies against MEST (Biorbyt, Cambridge, UK), E-cadherin (BDibodies use, San Jose, CA, USA, AB_397581), RASAL1 (Invitrogen, AB_2807382), actin (Santa Cruz Biotechnology, Santa Cruz, CA, USA, AB_626632), SRCIN1 (AB_2881315) and PURA (AB_2173875) from Proteintech, Flag (AB_259529) from Sigma–Aldrich, and p-ERK (AB_2315112), ERK (AB_390779), vimentin (AB_10695459), snail (AB_2889994), p-RAF (AB_2067317) and p-MEK (AB_331648) from Cell Signaling Technology.

### Homology modeling and molecular docking between MEST and PURA

Homologous proteins were identified by scanning the protein sequence of MEST or PURA against 3D structures deposited in protein data bank using PDB BLAST. The 1MJ5_A was found to be the best template structure for MEST, and the 3K44_B for PURA. The target and template structure (1MJ5_A and 3K44_B) were aligned using sequence alignment protocol. The 3D model of MEST and PURA were generated with MODELER protocol in Discovery Studio 4.5. Out of 10 models generated during this process, the best model based on the lowest DOPE (Discrete Optimized Protein Energy) score and PDF (Probability Density Function Energy) energy was selected. The ZDOCK^23^ in Discovery Studio 3.5 was used to dock PURA model to the MEST. The dockings were carried out in 5400 poses, screened out into 2000 better poses calculated by the ZRANK rescoring method. Given that the ZDock scores >15, ZRank scores <50 and the binding free energies, we chose the most suitable pose381(ZDock scores = 16.44, ZRank scores = −32.608) of these complexes. The pose with lowest energy of binding was extracted for further analysis (−80.6969 kcal mol−1 according to the MM-PBSA method^24^). Some residues of PURA contribute >0.5 kcal mol−1: Glu90, Gly95, Pro130, Asp131, Leu132, Gln134, Gln136, Pro139, Arg140, Lys160, Glu161, Gln163, Phe167, Arg169, Gln184 and Gln186. In addition, several residues of MEST contribute >0.5 kcal mol−1: Asp61, Val65, Val66, Glu70, Glu90, Leu94, His97, Gln137, Asn138, Arg139, Arg140, Asn142, Ile332 and Phe335. The importance of representative basic amino acids was proved using point alanine-scanning mutagenesis and immunoprecipitation experiments.

### Coimmunoprecipitation (co-IP)

Coimmunoprecipitation was performed as previously described.^25^ In brief, the cell lysates were prewashed with IgG (Santa Cruz Biotechnology, AB_737182) and protein A/G Sepharose beads (Invitrogen) for 1ruz Biotechnology, Val65, Val66, Glu70, Glu90, Leu94, His97, Gln137, Asn138, Arg139, Arg140, Asn142, Ile332, followed by incubation with protein A/G Sepharose beads for 4ad. The beads were washed and eluted for Western blot analysis.

### Chromatin immunoprecipitation (ChIP)-quantitative PCR

The ChIP assay was performed as previously described by using a simple ChIP enzymatic chromatin IP kit (Cell Signaling Technology) according to the manufacturer's manual.^6^ In brief, protein and DNA crosslinking was performed using 37% formaldehyde, followed by sonication and chromatin digestion. The protein-DNA complexes were immunoprecipitated using PURA antibody or negative control IgG antibody, and the purified DNA was subjected to qPCR analysis. Relative expression was calculated using the comparative Ct method after normalization to the GAPDH control.

### Molecular docking

AutoDock Vina was employed to screen the potential molecules binding to the MEST protein from the Chemdiv Database and Maybridge Database. During the docking process, semiflexible docking simulations were performed with the Lamarckian genetic algorithm, and the top 20 compounds with the best docking scores were obtained.

### Surface plasmon resonance (SPR)

The experiment was performed as previously described.^26^ SPR analysis was performed using the Biacore X100 system (GE Healthcare Life Sciences, Marlborough, MA, USA). The MEST protein was immobilized by amine coupling onto flow cell 2 of a CM7 chip (GE Healthcare Life Sciences). Following immobilization, the chip was washed for 30 min with PBS buffer. Small molecules in PBS buffer were passed over the chip at 30 μl/min for 90 s at 25 °C.

### Modified enzyme-linked immunosorbent assay (ELISA)

Modified ELISA was performed as described previously.^27^ In brief, after coating with the binding antibody against GST-tag (Proteintech), the 96-well plates were washed with PBS followed by blocking with 5% bovine serum albumin (BSA), incubation with the purified fusion protein PURA-GST for 5 h and purified MEST-His protein for 3 h. After the small molecules were added into each well, the plates were incubated with His antibody and the corresponding secondary antibody as well as tetramethylbenzidine (TMB), absorbance was measured, and the levels of MEST-PURA interactions were determined.

### *In vitro* cell migration and invasion assay

BioCoat Matrigel invasion chambers (BD Biosciences, Bedford, MA, USA) were used to compare the invasion of ESCC cells.^5^ The invasive activity of ESCC cells was evaluated with the use of an 8-μm pore-size invasion chamber coated with Matrigel. The cells suspended in serum-free medium were seeded into the upper compartment, and the lower chamber was filled with complete medium. The invaded cells were fixed in 100% methanol for 15 min and stained with 0.2% crystal violet for 5 min.

### Experimental metastasis model

The experiment was performed as previously described.^5^ Briefly, 1 × 10^6^ luciferase-expressing ESCC cells (KYSE150-Luc or KYSE150-Luc-LM5) expressing MEST and vector control or shMEST and shCON were injected intravenously into the animals via the tail vein. Metastasis was monitored weekly by bioluminescent imaging (Xenogen IVIS Lumina II *in vivo* imaging system, PerkinElmer, Hopkinton, MA, USA). All the animal experiments were approved by the Ethics Committee for Animal experiments of Jinan University (Number: 201872-11), and the mice were cared for under standard conditions according to institutional guidelines. Mice were euthanized under anesthesia at the end of studies.

### Multiorgan metastasis model

The experiment was performed as previously described.^6^ The ESCC cell line EC9706-Luc, which is capable of forming multiorgan metastasis (including lung, kidney and liver), was intravenously injected into the tail veins of NOD-Prkdc^em26Cd52^Il2rg^em26Cd22^ (NCG) mice (Nanjing Galaxy Biopharma, Nanjing, China), and metastasis was monitored weekly by bioluminescent imaging as described above.

### *In vivo* delivery of miR-449a

The miR-449a oligonucleotide or miR-CON (GenePharma, Shanghai, China) was formulated with a polymer-based agent (*in vivo*-jectPEI; Polyplus, Illkirch, France) according to the manufacturer's instructions.^17^ In the experiment involving systemic miR-449a treatment, the miR-449a or miR-CON oligonucleotide was formulated with a polymer-based agent (in vivo-jectPEI; Polyplus, Illkirch, France) according to the manufacturer's instructions and then intravenously injected into mice biweekly. miR-449a treatment was given five times in the multiorgan metastasis model, and three times in the lung metastasis model. Ten weeks after cell injection in the multiorgan metastasis model and six weeks after cell injection in the lung metastasis model, bioluminescent imaging was performed to observe the metastasis of cancer cells (Xenogen IVIS lumina II, PerkinElmer, MA). The signal was analyzed using Living Image R Software Version3.1.

### Hematologic analyses

In brief, ALT and AST levels in mouse serum were determined using commercial kits (HuiLi Biotech Ltd., Changchun, China). The amount or percentage of white blood cells (WBC), red blood cells (RBC), hemoglobin (HGB), platelets (PLT), neutrophils and lymphocytes were determined by hematologic analyzers (Mondrary, Shenzhen, China).

### Gene expression and survival data from public databases

Gene expression analysis was carried out as previously described.^5^ Based on the TCGA database or gene expression datasets downloaded from GEO, the normalized mRNA expression of MEST and miR-449a in clinical cancer specimens was compared with that in normal controls using Student'st-test.

### Patient-derived xenograft (PDX) and metastasis model

The PDX mouse model was established as described previously.^28^ In brief, fresh tumor tissues were obtained from ESCC patients with informed consent, cut into pieces of 1 mm^3^ tissues, subcutaneously inoculated into NOD-Prkdc^scid^-Il2rg^em1IDMO^ mice (Beijing IDMO Co., Ltd, Beijing, China) and then maintained by passaging from mouse to mouse. The human ESCC specimens were collected in accordance with the Declaration of Helsinki. Informed consent was obtained from each participant.

### Statistical analysis

All *in vitro* experiments and assays were repeated at least 3 times. The data are expressed as the mean ± SD and were compared by *t*-test. Survival analysis was performed by the Kaplan–Meier method with the log-rank test using the Statistical Package for the Social Sciences (SPSS) (SPSS Inc, Chicago, IL, USA). *P* values < 0.05 were considered significant for all experiments.

### Role of funders

The funders were not involved in the study design, data collection, data analysis, interpretation or writing of the manuscript.

## Results

### CRISPR/Cas9 screening and gene profiling identifies MEST as a driver of cancer metastasis

**Fig. 1:**
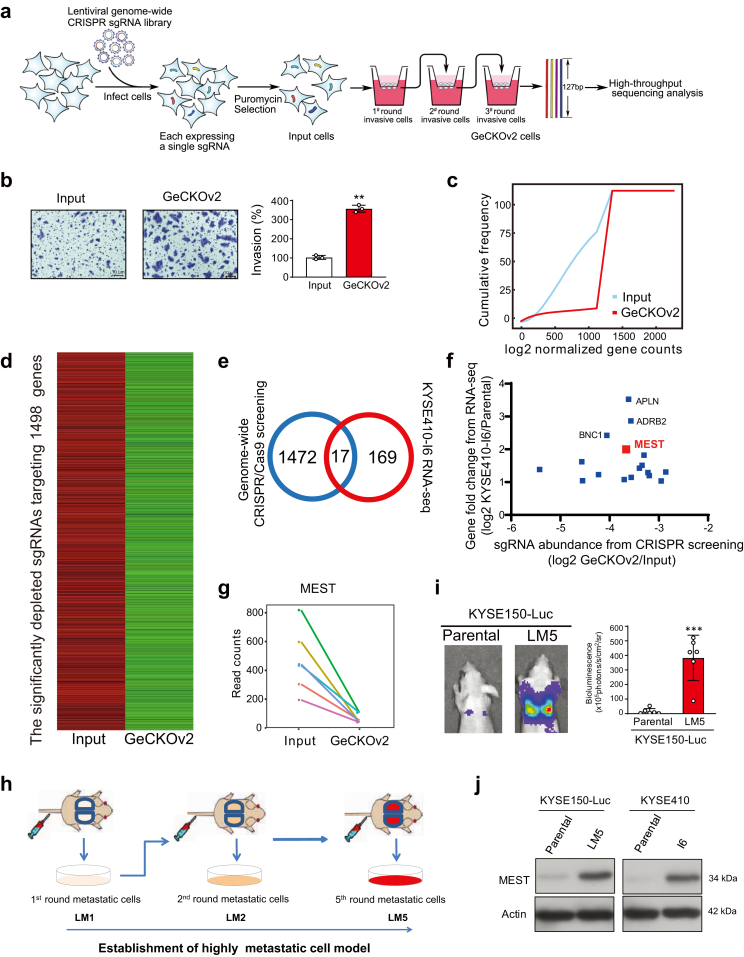
**Integrative analysis of genome-wide CRISPR/Cas9 screening and gene profiling of highly metastatic cell model identifies MEST as a driver of cancer metastasis.** (**a**) Schematic diagram illustrating the CRISPR/Cas9 screening of key regulators of cancer metastasis. (**b**) Comparison of invasion ability of GeCKOv2 cells and input cells after three rounds of invasion selection. (**c**) Cumulative frequency of sgRNAs in GeCKOv2 cells and input cells. (**d**) A heatmap displaying the 8927 significantly depleted sgRNAs targeting 1489 genes. (**e**) A total of 17 genes were overlapped between the data from genome-wide CRISPR/Cas9 screening and RNA-seq of highly invasive ESCC subline. (**f**) Scatter plot showing the sgRNA abundance from CRISPR/Cas9 screening and the expression fold change from RNA-seq of the 17 overlapped genes. (**g**) Comparison of read counts of individual sgRNAs targeting MEST in GECKOv2 and input cells. (**h, i**) Diagram illustrating the generation of highly metastatic cancer cells (KYSE150-Luc-LM5) by using a serial intravenous injection mouse model. **(j**) Western blot showing the expression of MEST in KYSE150-Luc-LM5 and KYSE150 cells, as well as KYSE410-I6 and KYSE410 cells. Bars, SD; ∗, P < 0.05; ∗∗, P < 0.01; ∗∗∗, P < 0.001, the student's *t* test.

A genome-wide screening was performed using genome-scale CRISPR knockout library (GeCKOv2) containing 123,411 sgRNAs targeting 19,050 protein-coding human genes (average of 6 sgRNAs/gene) and 7456 sgRNAs targeting 1864 microRNAs (miRNAs).^29^ We transduced ESCC cells KYSE150-Luc with the GeCKOv2 library at ∼500X coverage and an MOI of 0.3 to favor single viral integrations. After sgRNA-expressing cells were enriched by puromycin selection for one week, we divided the cells into two populations, one for control (Input) and the other for 3 rounds of invasion selection (GeCKOv2) using Matrigel-coated Boyden chambers. Genomic DNA was isolated from the two cell populations, and next-generation sequencing was used to measure read counts of each sgRNA (Fig. 1a). The GeCKOv2 cells exhibited enhanced invasion ability compared with input cells (Fig. 1b). Next-generation sequencing of genomic DNA revealed a markedly reduced diversity of sgRNAs in the GeCKOv2 cells (Fig. 1c), and 8927 significantly depleted sgRNAs targeting 1489 genes, inhibition of which potentially delayed cellular invasion ability, were identified (fold change of GeCKOv2/Input <0.5, *P* < 0.005; Fig. 1d and Supplementary Table S1).

In our previous study, we selected highly invasive ESCC cells (I3) via three rounds of selection with Boyden chambers.^5^ In the present study, we repeated the procedure three more times and generated more invasive cells, designated I6 cells, after which profiles of the differentially expressed genes were compared. Integrated analysis of the 1489 genes derived from the CRISPR/Cas9 screening and the 186 significantly upregulated genes in highly invasive cells (fold change of KSYE410-I6/Parental >2, FDR <0.001; Supplementary Table S2) generated a shortlist of 17 overlapped genes (Fig. 1e). Among the candidate genes, we were encouraged to see that the top 3 ranked genes, including basonuclin 1 (BNC1), β2 adrenergic receptor (ADRB2), and apelin (APLN), have been well reported as important regulators of cancer metastasis,^30^, ^31^, ^32^ which strongly suggests that our experimental models and selection strategies are useful in profiling metastasis-associated genes (Supplementary Fig. S1a and b; Supplementary Table S3).

MEST attracted our attention because it ranked 4th and more importantly, it has been reported to function as both tumor suppressor and oncogene.^33^^,^^34^ This dual role of MEST points to the need to better understand its biological function and action mechanism in human cancer (Fig. 1f). In addition, read counts for each MEST-targeted sgRNA were significantly decreased in the GeCKOv2 cells after three rounds of invasion selection (Fig. 1g). To better mimic the progression of metastasis, we further established highly metastatic cell lines via serial injection and selection (designated LM5) and verified that LM5 cells had stronger metastatic ability, as evidenced by bioluminescence imaging and histological analysis of the lungs (Fig. 1h and i and Supplementary Fig. S1c and d**)**. Western blot analysis confirmed increased MEST protein expression in both LM5 and I6 cells to a level observed in the parental cells (Fig. 1j), suggesting that MEST may be a key regulator of cancer metastasis.

### MEST upregulation is correlated with poor prognosis in esophageal cancer

**Fig. 2:**
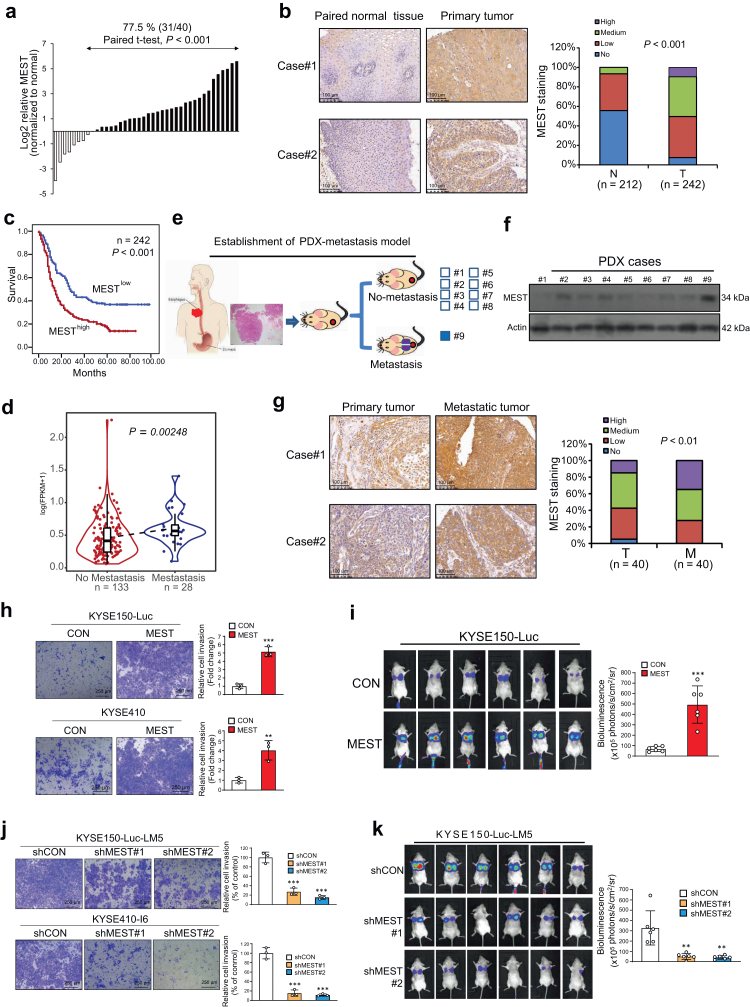
**Clinical and biological significance of MEST in ESCC metastasis.** (**a**) qRT-PCR analysis and expression pattern of MEST in 40 pairs of ESCC tumor and normal tissues. (**b**) Expression pattern of MEST in 242 ESCC tissues and 212 paired normal tissues. (**c**) Kaplan–Meier analysis curves of MEST expression in 242 ESCC patients. (**d**) Violin plot showing the expression of MEST in primary ESCC without (n = 133) or with metastasis (n = 28) in The Cancer Genome Atlas (TCGA). (**e, f**) Diagram showing the establishment of a patient-derived xenograft (PDX) metastasis model and comparison of MEST expression in the nine PDXs. (**g**) Expression pattern of MEST in 40 ESCC tumors and corresponding metastatic tissues. (**h**) Boyden chamber assay showing the effect of MEST on the invasive abilities of KYSE150 and KYSE410 cells. (**i**) Bioluminescence imaging and quantification showing that MEST-overexpressing ESCC cells had a higher potential to metastasize to the lungs in mice than control cells (n = 6/group). (**j**) Boyden chamber assay showing the effect of MEST knockdown on the invasion of ESCC cells. (**k**) Effect of MEST silencing on tumor metastasis (n = 6/group). Bars, SD; ∗, P < 0.05; ∗∗, P < 0.01; ∗∗∗, P < 0.001, the student's *t* test.

To examine the clinical significance of MEST dysregulation in esophageal cancer, we determined the expression of MEST in 40 ESCC tissues and paired normal tissues, and the qRT-PCR results showed the upregulation of MEST in the majority of ESCC cases (31/40 cases, 77.5%) (Fig. 2a). A tissue microarray (TMA) consisting of 242 primary ESCC tissues and 212 nontumor tissues was analyzed, and the expression of MEST was found to be higher in the tumor tissues than in the paired nontumor tissues (*P* < 0.001, the student's *t* test) (Fig. 2b and Supplementary Fig. S2a). Moreover, high MEST expression was significantly associated with lymph node metastasis in cancer patients (Pearson *χ*^2^ test, *P* < 0.001, the student's *t* test, Table 1). Kaplan–Meier survival analysis showed that the patients with high tumor MEST expression had significantly shorter survival (median survival = 13 months) than the patients with lower tumor MEST expression (median survival = 31 months) (log-rank test, *P* < 0.001, Fig. 2c). Data from The Cancer Genome Atlas (TCGA) were analyzed, and the results showed that MEST is highly expressed in patients with metastasis (n = 28) in comparison with patients without metastasis (n = 133) (two-sided Wilcoxon rank-sum test, *P* = 0.00248, Fig. 2d). Here, a patient-derived xenograft (PDX) model was generated by implanting tumor specimens from 9 ESCC patients into immunodeficient mice. Interestingly, MEST was found to be highly expressed in the case with metastasis in comparison with the nonmetastatic cases (Fig. 2e and f). Furthermore, we detected the expression of MEST in another independent tissue microarray consisting of 40 pairs of primary tumors and metastatic tissues, and the results demonstrated that the expression of MEST in metastatic tissues was higher than that in primary tumors (*P* < 0.01, the student's *t* test) (Fig. 2g). Analysis of data from TCGA datasets indicated that MEST expression was frequently upregulated in tumor tissues compared with nontumor tissues in multiple cancer types, including esophageal, bladder, colon, liver, and lung cancers (Supplementary Fig. S2b). In public databases, high MEST expression was found to be associated with poor prognosis in many cancer types, including brain, cervical, kidney and liver cancers (Supplementary Fig. S2c). Taken together, these results suggest that MEST may be a useful biomarker for cancer diagnosis and prognosis.

**Table 1:** Correlation between MEST expression levels and clinicopathological parameters in 242 cases of esophageal cancer.

Variable	n	Low Mest	High Mest	*P* value
Age (years)				
≤55	47	23	24	
>55	195	97	98	1.000
Sex				
Female	58	31	27	
Male	184	89	95	0.499
T-Stage				
1/2	46	29	17	
3/4	179	82	97	**0.037∗**
N-Stage				
N0	115	69	46	
N1/N2/N3	124	49	75	**0.001∗∗**
M-Stage				
M0	238	117	121	
M1	4	3	1	0.305
Grade				
I & II	187	91	96	
III & IV	55	29	26	0.591
Bold values indicate significant differences. ∗*P* < 0.05; ∗∗*P* < 0.01; Pearson Correlation Analysis.				

### MEST promotes esophageal cancer invasion and metastasis

We next investigated the biological significance of MEST in cancer progression. In gain-of-function studies, the Boyden chamber assay showed that overexpression of MEST significantly increased the invasive ability of ESCC cells without influencing proliferation (Fig. 2h and Supplementary Fig. S2e). The results from the *in vivo* experiment indicated that intravenously injected MEST-overexpressing ESCC cells had a higher potential to metastasize to the lungs in mice (Fig. 2i and Supplementary Fig. S2e). In contrast, the opposite results were observed in MEST-knockdown KYSE150-Luc-LM5 and KYSE410-I6 cells *in vitro* and *in vivo* (Fig. 2j and k and Supplementary Fig. S2f). Furthermore, we noted that overexpression of MEST induced elevated expression of the mesenchymal markers vimentin and snail, as well as downregulation of the epithelial marker E-cadherin, while the opposite pattern was observed with MEST knockdown (Supplementary Fig. S2g). These results suggest that MEST functions in invasion and metastasis by inducing epithelial–mesenchymal transition (EMT).

### SRCIN1-RASAL1/ERK-snail signaling mediates the effects of MEST on tumor metastasis

Since MEST has been reported to be related with lipid metabolism,^35^ the levels of free fatty acids and triglyceride were detected in ESCC cells with MEST overexpression. As shown in Supplementary Fig. S3a, MEST did not affect the levels of free fatty acids or triglyceride in ESCC cells. In addition, we constructed plasmid expressing MEST mutant (D147A), in which the hydrolase site of MEST was mutated^36^ and Boyden chamber assay was performed to compare the invasion ability between the ESCC cells overexpressing wild-type MEST and mutant MEST, respectively. The results indicated that MEST enhanced the invasion of ESCC, and this biological function was not related to its hydrolase affect (Supplementary Fig. S3b).

**Fig. 3:**
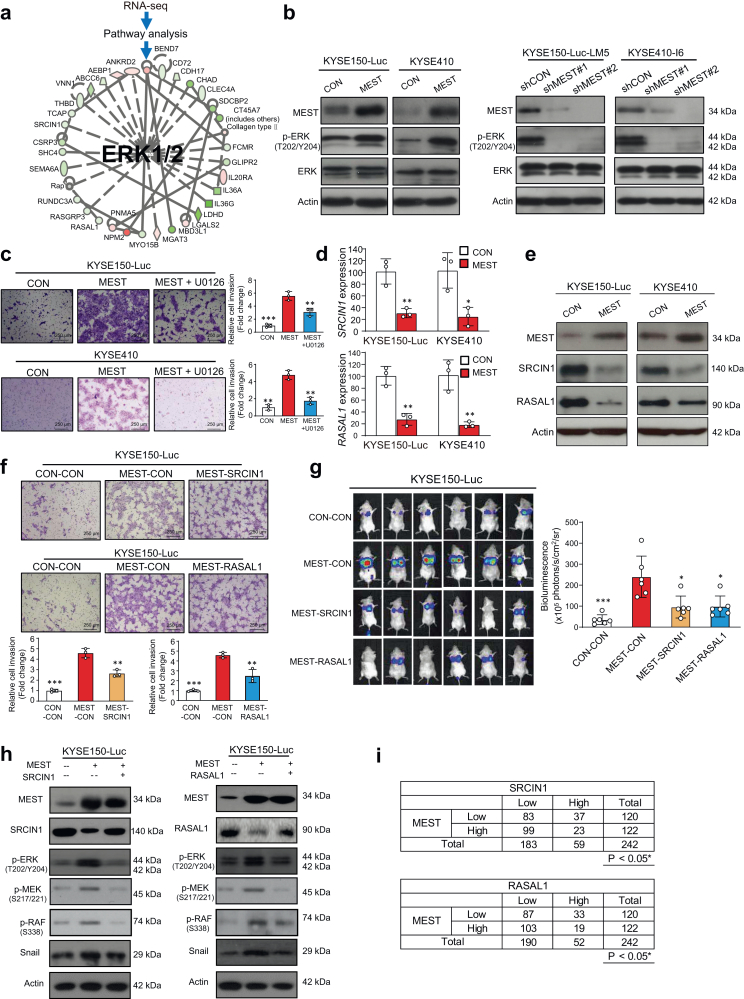
**MEST activates ERK signaling through the regulation of SRCIN1 and RASAL1 expression.** (**a**) IPA analysis showing that MEST may activate the ERK signaling pathway. (**b**) Western blot showing the expression of p-ERK and ERK when the expression of MEST was manipulated. (**c**) Boyden chamber showing the effect of MEST on ESCC invasion in the presence or absence of the MEK inhibitor U0126 (10 μM) for 24 h. (**d, e**) The mRNA and protein expression of SRCIN1 and RASAL1 upon ectopic expression of MEST in KYSE150 and KYSE410 cells. (**f**) Boyden chamber assay showing the invasion of MEST-overexpressing cells when the expression levels of SRCIN1 and RASAL1 were manipulated. (**g**) Bioluminescence imaging and quantification showing the metastatic potential of KYSE150-Luc-MEST-CON, KYSE150-Luc-MEST-SRCIN1, KYSE150-Luc-MEST-RASAL1, and vector control cells (n = 6/group). (**h**) Western blot showing the expression of p-ERK, p-MEK, p-RAF and snail in MEST-overexpressing cells when the expression levels of SRCIN1 and RASAL1 were manipulated. (**i**) Association between MEST and SRCIN1 or RASAL1 in ESCC. Bars, SD; ∗, P < 0.05; ∗∗, P < 0.01; ∗∗∗, P < 0.001, the student's *t* test.

Although some studies found that MEST can promote cancer metastasis in lung cancer and breast cancer,^37^^,^^38^ the role of MEST in ESCC has not been reported. To explore the mechanisms of action underlying the role of MEST in cancer metastasis, RNA sequencing (RNA-seq) was used to compare the gene profiles between MEST-overexpressing cells and vector control cells. Ingenuity pathway analysis (IPA) of differentially expressed genes suggested that the ERK signaling pathway may be involved in the biological function of MEST (Fig. 3a). The Western blot results confirmed that overexpression of MEST enhanced ERK phosphorylationing the role of MEST in cancer metastasis, RNA sequencing (RNA-seq) was used to compare the gene profiles between MEST-o-I6 cells (Fig. 3b). Boyden chamber assays showed that the addition of the MEK signaling inhibitor U0126 markedly abrogated MEST-induced cancer invasion (Fig. 3c).

Among a cluster of differentially expressed genes that constitute the signaling network with ERK as the central hub, SRC kinase signaling inhibitor 1 (SRCIN1) and RAS protein activator like 1 (RASAL1) drew our great attention. SRCIN1 has been reported to block ERK signaling,^39^ and RASAL1 can act as a tumor suppressor gene in gastric cancer through inactivation of the ERK pathway.^40^ Our experimental data indicated that overexpression of MEST can decrease the expression of SRCIN1 and RASAL1 at both the mRNA and protein levels (Fig. 3d and e and Supplementary Table S3**)**. In addition, we demonstrated that knockdown of MEST significantly increased expression levels of SRCIN1 and RASAL1 in KYSE150-luc-LM5 and KYSE410-I6 cell lines (Supplementary Fig. S3c), which leading us to speculate that SRCIN1 and RASAL1 may mediate the function of MEST in cell invasion and metastasis. This hypothesis was confirmed by our experimental results showing that ectopic expression of SRCIN1 or RASAL1 can abolish the effect of MEST on cell invasion, RAF-MEK-ERK phosphorylation and snail expression (Fig. 3f–h). In addition, we obtained consistent results in the loss-of-function study (Supplementary Fig. S4a and b). Furthermore, since the clinical relevance of SRCIN1 and RASAL1 in ESCC remains unknown, immunohistochemical analyses of SRCIN1 and RASAL1 were performed in the same tissue microarray as above. The results demonstrated significantly lower expression of SRCIN1 and RASAL1 in the tumors than in the paired normal tissues (*P* < 0.001, the student's *t* test) (Supplementary Fig. S4c–h, Supplementary Tables S4 and S5**)** and, more importantly, a negative correlation between the expression of MEST and SRCIN1, as well as MEST and RASAL1 (Pearson *χ*^2^ test, *P* < 0.05) (Fig. 3i), corroborating our findings above on the important role of MEST in the regulation of SRCIN1 and RASAL1. These data collectively demonstrate that SRCIN1 and RASAL1 are crucial for the function of MEST in regulating ERK signaling and cancer metastasis.

### MEST interacts with PURA to activate the ERK signaling pathway

**Fig. 4:**
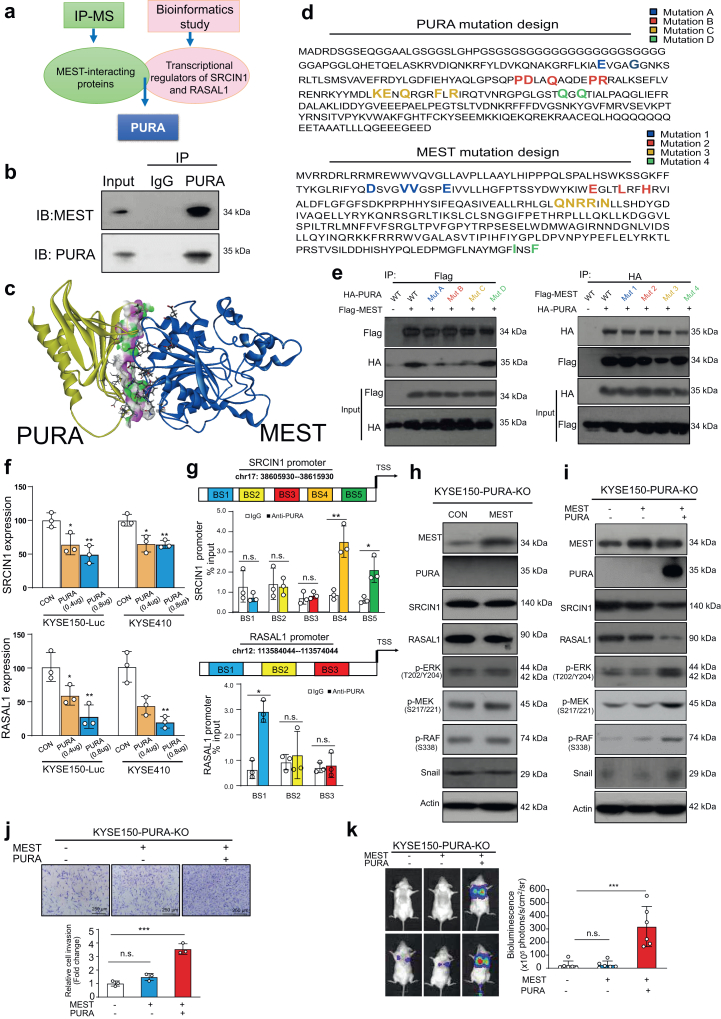
**MEST interacts with PURA to activate the ERK-snail pathway.** (**a**) Diagram showing the approach to identify the overlapping proteins that interact with MEST and simultaneously regulate the transcription of SRCIN1 and RASAL1. (**b**) Coimmunoprecipitation showing the binding of PURA with MEST. (**c**) 3D modeling showing the interaction between MEST and PURA. (**d**) Diagram showing the key amino acids selected to investigate the interaction between MEST and PURA, as well as the mutation design for expressing mutant proteins. (**e**) The interaction between PURA and wild-type or mutant MEST, as well as MEST and wild type or mutant PURA in KYSE150-Luc cells. (**f**) qRT-PCR assay showing the effect of PURA overexpression on the mRNA expression levels of SRCIN1 and RASAL1 in KYSE150 and KYSE410 cells. (**g**) Putative binding sites of PURA in the promoter regions of SRCIN1 and RASAL1 were identified by *in silico* prediction, and the enrichment of PURA in the promoter regions was determined by ChIP. (**h, i**) Western blot showing the effect of MEST overexpression on the expression levels of SRCIN1, RASAL1, p-ERK, p-MEK, p-RAF and snail in PURA-deficient ESCC cells with or without reintroduction of PURA. (**j, k**) Boyden chamber and experimental metastasis assays were performed to examine the invasive and metastatic potential of PURA-deficient ESCC cells when both MEST and PURA or MEST alone were overexpressed. Bars, SD; ∗∗, P < 0.01; ∗∗∗, P < 0.001, the student's *t* test.

To decipher the molecular mechanisms by which MEST regulates the SRCIN1/RASAL1-mediated ERK pathway, immunoprecipitation coupled with liquid chromatography tandem mass spectrometry (IP-MS) was performed to identify the interacting partners of MEST (Supplementary Table S6). Meanwhile, a list of putative transcription factors of SRCIN1 and RASAL1 was obtained by using the TcoF-DB v2 database^41^ (Fig. 4a). Among the overlapping proteins identified, purine rich element binding protein A (PURA), which was confirmed to interact directly with MEST in ESCC cells by our coimmunoprecipitation (co-IP) assay (Fig. 4b), became our research focus. The Western blot results showed that PURA overexpression induced the downregulation of SRCIN1 and RASAL1, activation of the ERK signaling pathway and upregulation of snail. Conversely, PURA knockdown with the siRNA approach generated the opposite results **(**Supplementary Fig. S5a).

First, to further elucidate the domains responsible for the interaction between MEST and PURA, a 3D model of the MEST and PURA proteins was generated by homologous modeling (Fig. 4c). Molecular mechanics Poisson-Boltzmann surface area (MM-PBSA) was applied to calculate the binding free energy between the MEST and PURA, then alanine mutation scanning was performed in the binding sites of MEST-PURA. With the output of MM/PBSA calculations and alanine mutation scanning, we constructed 4 plasmids expressing different PURA mutants to determine which amino acids are the key binding sites (Fig. 4d). The MEST-expressing plasmid was cotransfected into ESCC cells with the plasmid expressing wild-type (WT) or mutant (Mut) PURA, and the results showed that the interaction between MEST and PURA was strongly inhibited when site#A, site#B or site#C was mutated, indicating the critical role of these amino acids in the MEST-PURA interaction (Fig. 4e, left panel). We also performed a similar experiment in the opposite way, and the co-IP data from ESCC cells cotransfected with PURA and wild-type or mutant MEST plasmids suggest the critical role of site#3 (aa138-142) in MEST in its binding with PURA (Fig. 4e, right panel).

Second, the qRT-PCR results showed that PURA negatively regulated the expression of SRCIN1 and RASAL1 at the mRNA level (Fig. 4f and Supplementary Fig. S5b), leading us to propose that PURA may bind to the promoters of SRCIN1 and RASAL1. A chromatin immunoprecipitation assay (ChIP) was used to determine whether there is a physical interaction between the PURA protein and the promoter regions of SRCIN1 and RASAL1. On the one hand, we found that of the five potential PURA binding sites in the promoter region of SRCIN1 (designated BS1, BS2, BS3, BS4 and BS5), only the BS4 and BS5 fragments were enriched in the PURA immunoprecipitates. On the other hand, only the BS1 fragment of the three putative binding sites in the RASAL1 promoter region responded to PURA immunoprecipitation (Fig. 4g). To further confirm the interaction between PURA and the promoters of SRCIN1 and RASAL1, site-specific mutations were generated, and luciferase assay data indicated that the binding sites validated above, namely, BS4 and BS5 in the SRCIN1 promoter and BS1 in the RASAL1 promoter, function as PURA-responsive elements **(**Supplementary Fig. S5c, Supplementary Table S7**)**.

To further study the essential role of PURA in the regulation of the SRCIN1/RASAL1-ERK-snail signaling pathway by MEST, CRISPR/Cas9 system was used to establish PURA-knockout (PURA-KO) cells (Supplementary Fig. S5d, Supplementary Table S8). MEST was overexpressed in PURA-deficient cells to determine the significance of PURA in the functional role of MEST in ESCC. Western blot data showed that ectopic MEST expression did not affect the expression levels of SRCIN1, RASAL1, p-RAF, P-MEK, p-ERK or snail in PURA-KO cells (Fig. 4h), whereas re-overexpression of PURA effectively rescued the effect of MEST on the downstream pathway (Fig. 4i). More importantly, unlike that in PURA-expressing cell lines, there was no change in the invasive and metastatic potential upon overexpression of MEST in PURA-deficient ESCC cells, and the promoting effects of MEST in cancer invasion and metastasis could be restored by re-overexpression of PURA (Fig. 4j and k). In addition, we found that MEST could enhance the transcriptional regulation of PURA on SRCIN1 and RASAL1 (Supplementary Fig. S5e and f). Taken together, our results demonstrate that MEST regulates SRCIN1/RASAL1-ERK-snail signaling and cancer metastasis in a PURA-dependent manner.

### Frequent promoter hypermethylation and clinical significance of miR-449a in esophageal cancer

**Fig. 5:**
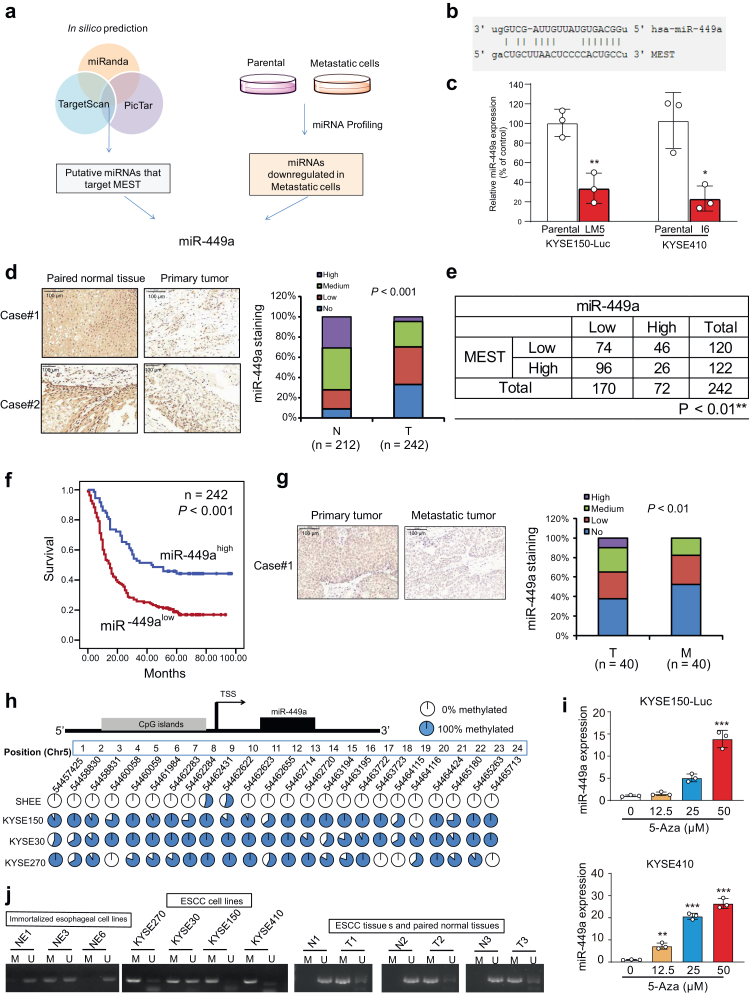
**Promoter hypermethylation results in deregulation of miR-449a in ESCC.** (**a**) Diagram showing the strategy to screen potential miRNAs that target MEST and inhibit cancer metastasis. (**b**) TargetScan bioinformatics algorithm showing the base pairing between miR-449a and the 3′UTR of MEST. (**c**) Comparison of miR-449a expression in KYSE150-Luc-LM5 or KYSE410-I6 cells with that in their respective parental cells by TaqMan miRNA PCR assay. (**d**) Representative image showing the expression of miR-449a in ESCC tumor and matched normal tissue (left panel). Expression pattern of miR-449a in primary tumor (n = 242) and matched normal tissue (n = 212) (right panel). (**e**) The correlation between MEST and miR-449a expression in 242 ESCC tumors examined is shown. (**f**) Kaplan–Meier analysis of overall survival of 242 ESCC patients stratified according to tumor miR-449a expression. (**g**) Representative image and expression pattern of miR-449a in primary ESCC tumor tissues (n = 40) and matched metastatic tissues (n = 40). (**h**) The methylation status of the miR-449a promoter in immortalized normal esophageal epithelial cell lines and ESCC cell lines was examined by whole genomic methylation sequencing. Each plot represents the methylation percentage of each site. (**i**) qRT-PCR analysis was used to detect the expression level of miR-449a in KYSE150 and KYSE410 cells treated with 5′-aza-2'deoxycytidine (5-Aza). (**j**) Detection of promoter hypermethylation in immortalized normal esophageal epithelial cell lines, ESCC cell lines and matched tumor tissues and nontumor tissues by methylation-specific PCR. M: methylated allele; U: unmethylated alleles. Bars, SD; ∗∗, P < 0.01; ∗∗∗, P < 0.001, the student's *t* test.

To reveal the upstream regulatory mechanisms of MEST, we initiated a screening for candidate miRNAs that can directly target MEST to regulate cancer metastasis. On the one hand, three miRNA target prediction software programs were utilized to search for the potential miRNAs that may bind to the 3′UTR of MEST. On the other hand, miRNA profiles were compared in highly metastatic ESCC cells and parental cells (Fig. 5a). miR-449a was found to satisfy the criteria of having seed regions that perfectly matched the 3′UTR of MEST and was downregulated in KYSE150-Luc-LM5 cells (Fig. 5b). Moreover, our CRISPR/Cas9 screening data also suggested that miR-449a may exert a suppressive function in cancer metastasis. A TaqMan qRT-PCR assay was performed, and lower miR-449a expression was observed in KYSE150-Luc-LM5 cells and KYSE410-I6 cells, as expected (Fig. 5c). The clinical relevance of miR-449a in ESCC remains to be elucidated. The expression of miR-449a in 40 pairs of ESCC tissues and matched normal tissues was determined by TaqMan qRT-PCR, and as shown in Supplementary Fig. S6a**,** miR-449a expression was not only significantly downregulated in tumor tissues compared with normal tissues but also negatively correlated with the expression of MEST (Supplementary Fig. S6b). *In situ* hybridization (ISH) was performed to determine the expression of miR-449a in the same tissue microarray shown in Fig. 2f, which consisted of 212 primary tumors and matched 242 nontumor tissues. The results indicated that the expression of miR-449a was significantly lower in the tumor tissues than in the paired normal tissues and correlated with tumor size (Pearson χ^2^ test, *P* < 0.05) and lymph node metastasis (Pearson χ^2^ test, *P* < 0.001) (Fig. 5d, Supplementary Fig. S6c and Supplementary Table S9). Moreover, miR-449a expression was significantly negatively associated with the expression level of MEST (Fig. 5e), supporting our hypothesis on the regulation of MEST by miR-449a. Kaplan–Meier survival analysis suggested that the patients with low tumor miR-449a expression had significantly shorter survival (median survival = 16 months) than the patients with high tumor miR-449a expression (median survival = 51 months) (log-rank test, *P* < 0.001, Fig. 5f). Next, we detected the expression of miR-449a in another tissue microarray, as shown in Fig. 2h, which consisted of 40 paired primary tumors and metastatic tissues. As indicated in Fig. 5g, miR-449a expression was markedly lower in metastatic tissues than in primary tumors (*P* < 0.01, the student's *t* test). By analyzing Gene Omnibus Express (GEO) datasets, we observed the downregulation of miR-449a in the majority of tumor tissues compared with nontumor tissues in multiple cancer types, including esophageal, prostate, breast, colon and gastric cancers (Supplementary Fig. S6d). These data collectively suggest that miR-449a may be a diagnostic and prognostic biomarker.

Abnormal epigenetic regulation contributes to the silencing of tumor suppressor protein-coding and noncoding genes, which may lead to diseases such as cancer. Next, whole-genome methylation sequencing was carried out in an immortalized normal esophageal epithelial cell line (SHEE) and three ESCC cell lines (KYSE150, KYSE30 and KYSE270). Interestingly, the CpG islands within the miR-449a promoter were heavily hypermethylated in the three ESCC cell lines compared with the immortalized normal esophageal epithelial cells (Fig. 5h, Supplementary Table S10**)**. KYSE150 and KYSE410 cells were treated with a DNA methylation inhibitor, 5′-aza-2'deoxycytidine (5-Aza), and a dose-dependent increase in the expression level of miR-449a was observed (Fig. 5i). Furthermore, methylation-specific PCR using methylation- or unmethylation-specific primers was also performed in ESCC cell lines and tissues (Supplementary Table S11). Methylated alleles were found in all four ESCC cell lines and three tumors. In contrast, unmethylated alleles were observed in all three immortalized normal esophageal epithelial cell lines (NE1, NE3 and NE6) and three normal tissues (Fig. 5j). These findings suggest that promoter hypermethylation largely accounts for the mechanism of miR-449a downregulation in ESCC.

### miR-449a targets MEST to inhibit cancer invasion and metastasis

**Fig. 6:**
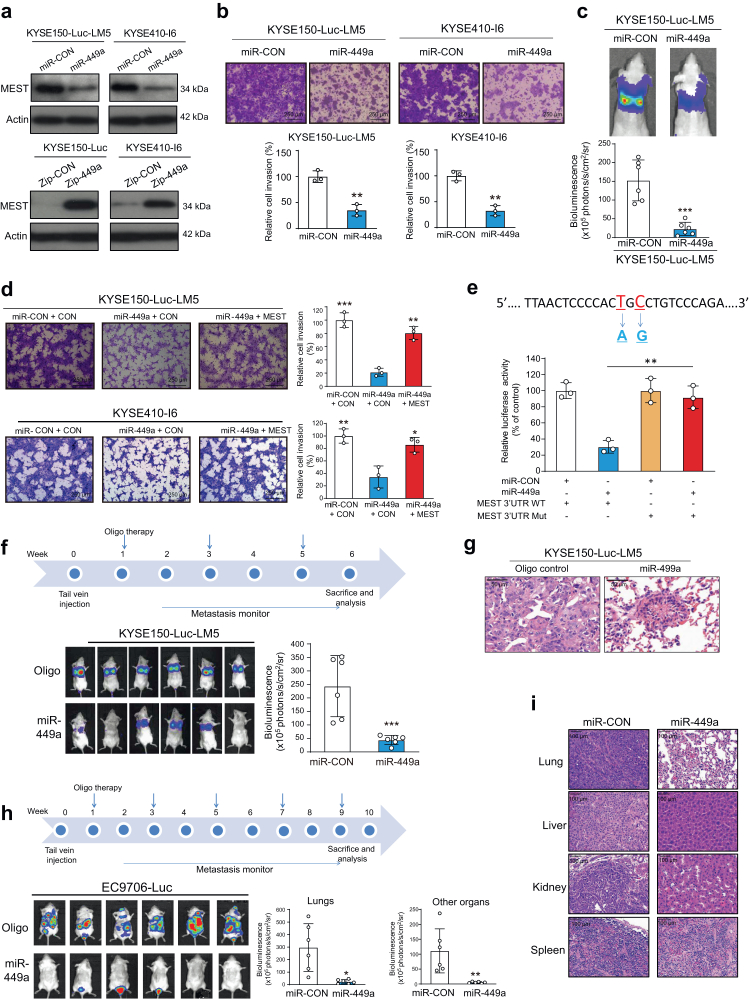
**miR-449a targets MEST to inhibit cancer invasion and metastasis.** (**a**) MEST expression was determined by Western blotting in ESCC cells with stable overexpression or knockdown of miR-449a. (**b, c**) Boyden chamber and bioluminescence imaging showing the effect of miR-449a overexpression on tumor invasion and metastasis. (**d**) Ectopic expression of MEST abolished the suppressive effect of miR-449a on ESCC cell invasion and metastasis. (**e**) Diagram illustrating the site mutations introduced in the reporter plasmid containing the MEST 3′UTR. The lower panel shows the luciferase activity of ESCC cells cotransfected with miR-449a and wild type or mutant MEST 3′UTR. (**f**) Experimental scheme and bioluminescence imaging showing the effect of systemically delivered 20 μg miR-449a oligonucleotide on tumor metastasis (n = 6/group). (**g**) H&E staining showing the metastatic niches in the lung tissues (n = 3). (**h**) The effect of miR-449a delivery on tumor metastasis was monitored in the multiorgan metastasis model (n = 6/group). (**i**) H&E staining showing the metastasis niches in multiple organs, including the lung, liver, kidney and spleen (n = 3). Bars, SD; ∗∗, P < 0.01; ∗∗∗, P < 0.001, the student's *t* test.

Next, the biological function of miR-449a in cancer progression was investigated. Western blot analysis showed that overexpression of miR-449a significantly reduced MEST expression in KYSE150-Luc-LM5 and KYSE410-I6 cells, whereas stable knockdown of miR-449a had the opposite effect on the expression of MEST (Fig. 6a). Gain- and loss-of-function experiments using a miR-449a mimic and inhibitor confirmed the regulation of MEST by miR-449a (Supplementary Fig. S7a). To examine the function of miR-449a in tumor invasion, which is still unknown, miR-449a was overexpressed in KYSE150-Luc-LM5 cells, with cells expressing miR-CON as control. As indicated in Fig. 6b and Supplementary Fig. S7b, overexpression of miR-449a led to a decrease in cell invasion; conversely, knockdown of miR-449a markedly enhanced the invasive potential of ESCC cells. Experimental metastasis assays and bioluminescent imaging demonstrated that ectopic miR-449a expression resulted in a delay in metastasis, whereas miR-449a silencing exerted the opposite effect (Fig. 6c and Supplementary Fig. S7c). In addition, a negative regulation of p-ERK, snail, and vimentin by miR-449a, as well as a positive regulation of SRCIN1, RASAL1, and E-cadherin, was observed (Supplementary Fig. S7d).

To investigate whether MEST mediates the effect of miR-449a on tumor invasion, we further enforced MEST expression in the miR-449a-overexpressing cell lines to establish KYSE150-Luc-LM5-miR-449a-MEST and KYSE410-I6-miR-449a-MEST cells, respectively, with vector as the control. Boyden chamber assays and Western blot analysis showed that the inhibitory effect of miR-449a on cancer cell invasion could be abrogated by overexpression of MEST (Fig. 6d and Supplementary Fig. S7e). To confirm whether MEST is directly regulated by miR-449a, the wild type and mutant fragments of the MEST 3 ts type a introduced into a luciferase reporter plasmid. As indicated in Fig. 6e and Supplementary Fig. S7f, miR-449a significantly decreased the luciferase activity of the wild-type MEST 3e further enforced MEST expressivity of the mutant MEST 3′-UTR, indicative of direct binding and regulation between miR-449a and the MEST 3′-UTR (Supplementary Table S4). Taken together, these results suggest that MEST may function as a key mediator in the suppressive effect of miR-449a in cancer invasion and metastasis.

### Systemically delivered miR-449a mimic suppresses tumor metastasis

Given the functional role of miR-449a in tumor metastasis, we explored the therapeutic efficacy of miR-449a in a preclinical setting. Our results showed that systemic delivery of a miR-449a oligonucleotide led to a significant decrease in lung metastasis compared with the vehicle, as evidenced by both bioluminescent imaging (Fig. 6f) and histological analysis of the lungs (Fig. 6g). The body weights of the mice were monitored, and no significant change was observed among the groups (Supplementary Fig. S8a). In addition, the hematologic analysis did not show any overt change in the levels of serum alanine transaminase (ALT) or aspartate transaminase (AST) or blood cell counts of nude mice (Supplementary Fig. S8b).

We further established a multiorgan metastasis mouse model.^42^ The results showed that injection of EC9706-Luc cells induced multiorgan metastasis in mouse lungs, kidneys and livers, whereas treatment with the miR-449a oligonucleotide led to a significant reduction in metastasis (Fig. 6h and i). These data collectively suggest the potential application of miR-449a oligonucleotides in cancer treatment without significant side effects.

### Identification of G699-0288 to inhibit the MEST-PURA interaction and cancer metastasis

**Fig. 7:**
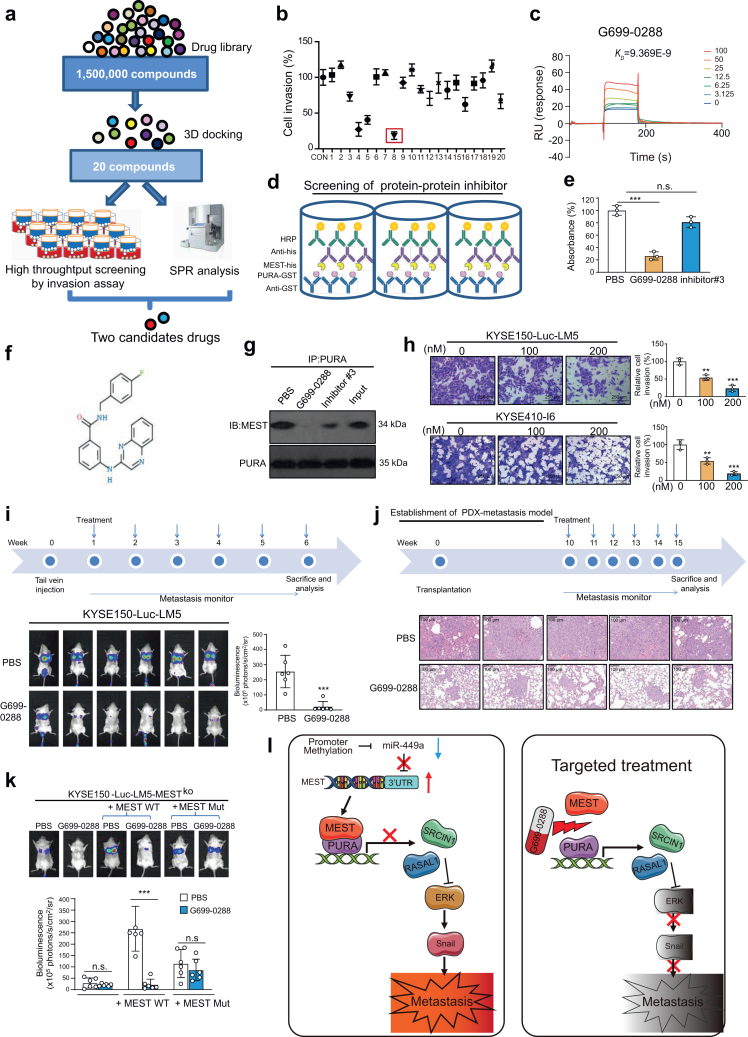
**Identification of G699-0288 as a lead compound to disrupt the MEST-PURA interaction and cancer metastasis.** (**a**) Diagram showing the approach to screen candidate compounds targeting MEST to suppress cancer metastasis. (**b**) The top 20 compounds were used to treat KYSE410-Luc-I6 cells, and the inhibitory effect on cell invasion was examined by the Boyden chamber assay. (**c**) SPR analysis revealing the binding of G699-0288 to the MEST protein. (**d**) The workflow of the modified ELISA screening system. In brief, the interaction between the recombinant proteins PURA-GST and MEST-His in the presence or absence of inhibitors was analyzed. (**e**) The G699-0288 compound, but not the inhibitor#3, significantly blocked the binding of MEST with PURA protein. (**f**) The structure of the G699-0288 compound. (**g**) A co-IP assay was performed to determine the interaction of MEST and PURA in the absence or presence of inhibitors. (**h**) Boyden chamber assay showing the effect of G699-0288 on ESCC cell invasion. (**i**) Bioluminescence imaging and quantification of lung metastasis in mice intravenously injected with KYSE150-Luc-LM5 cells and treated with G699-0288 (5 mg/kg). (**j**) Experimental scheme of the PDX metastasis model and microscopic images of the dissected lungs from the mice treated with G699-0288 and vehicle. (**k**) Experimental scheme illustrating the animal experimental design. Bioluminescence imaging and quantification of lung metastasis when wild type or mutant MEST protein was re-overexpressed in MEST-deficient cells in the presence or absence of G699-0288 (5 mg/kg) treatment (n = 6/group) (**l**). Schematic diagram summarizing the MEST-PURA as a key mechanism of cancer metastasis, which can be blocked by G699-0288. Bars, SD; ∗∗, P < 0.01; ∗∗∗, P < 0.001, the student's *t* test.

We aimed to identify small molecules that could directly target the MEST protein and simultaneously suppress cancer invasion and metastasis. Molecular docking was performed using amino acids 138–142 of MEST as a target. The top 20 compounds (Supplementary Table S13) with the best scores were selected for further screening, which combined a high-throughput invasion chamber assay with surface plasmon resonance (SPR) analysis (Fig. 7a–c and Supplementary Fig. S9 and b). Furthermore, a modified enzyme-linked immunosorbent assay (ELISA), which has been used in our previous study to screen small molecules targeting protein–protein interactions, was performed here to narrow the list of candidate compounds targeting the MEST-PURA interaction to a lead compound (named G699-0288) (Fig. 7d–f). A Co-IP assay was performed in the absence or presence of potential inhibitors, and results confirmed that compound G699-0288 markedly reduced the interaction of MEST and PURA protein (Fig. 7g). Next, the effect of G699-0288 on the SRCIN1/RASAL1-ERK-snail signaling pathway was investigated by Western blotting (Supplementary Fig. S9c). Additionally, the results showed that neither p-AKT or p-GSK3β could be affected by G699-0288, further supporting the hypothesis that G699-0288 exerts anticancer effect in a MEST-dependent manner (Supplementary Fig. S9d). The *in vitro* experiments were performed, and G699-0288 was found to suppress cancer cell invasion (Fig. 7h). The effect of G699-0288 on tumor metastasis was demonstrated in a tail vein injection model (Fig. 7i and Supplementary Fig. S9e) and a PDX metastasis model (Fig. 7j). More importantly, compared with the inhibitory rate of selumetinib (81.1%), G699-0288 suppressed tumor metastasis more significantly with an inhibitory rate of 93.4% **(**Supplementary Fig. S9f**)**. The histological and blood biochemistry data consistently suggested the low toxicity of G699-0288 **(**Supplementary Fig. S9g and h**)**.

Next, to investigate the target engagement of G699-0288, the MEST-knockout cell line (KYSE150-Luc-LM5-MEST-KO) was established by using CRISPR/Cas9 technology (Supplementary Fig. S9i). The invasive potential of KYSE150-Luc-LM5-MEST-KO, KYSE150-Luc-LM5-MEST-KO-WT or KYSE150-Luc-LM5-MEST-KO-mut cells in presence or absence of G699-0288 was compared. A significantly inhibitory effect of G699-0288 on invasion ability was observed in KYSE150-Luc-LM5-MEST-KO-WT cells, but not in KYSE150-Luc-LM5-MEST-KO or KYSE150-Luc-LM5-MEST-KO-mut cells (Supplementary Fig. S9j). We next confirmed this finding in animal models, and noted that in the mice intravenously injected with KYSE150-Luc-LM5-MEST-KO cells, treatment with G699-0288 did not suppress cancer metastasis, whereas re-expression of wild type MEST rescued the inhibitory effect of G699-0288 on lung metastasis. We did not observe a restored effect when the cells were reinduced with mutant MEST (Fig. 7k).

## Discussion

Although advances in clinical therapy have improved the survival of patients with localized ESCC, the high mortality of ESCC is mainly attributed to tumor metastasis.^43^ Genome-wide CRISPR/Cas9 screening is a powerful tool for unbiased discovery and functional characterization of genetic alterations leading to the phenotype of interest.^4^^,^^44^ In this study, we identified the MEST protein as an important regulator of tumor metastasis in ESCC. MEST is known to play an important role in multiple biological processes, including human adipogenesis and muscle regeneration.^45^^,^^46^ However, the role of MEST in cancer has not been reported till now. Here, our study revealed that MEST is a promising predictor for the prognosis of ESCC patients in the clinic. More importantly, gain- and loss-of-function experiments demonstrated that MEST could promote invasion and metastasis *in vitro* and *in vivo* through activation of the ERK signaling pathway. However, the diverse functions and mechanisms of MEST in ESCC proliferation as well as other cancers need further studies.

Targeting the MAPK-ERK pathway has long been considered a promising strategy for cancer therapy.^47^ SRCIN1 was identified as a tumor suppressor gene that plays a major role in Src inactivation and regulation of the RAS-ERK pathway.^48^ RASAL1, a member of the RAS GAP family,^49^^,^^50^ has been reported to act as a major tumor suppressor gene that influences the proliferation and invasion of cancer cells by regulating the RAS/ERK signaling pathway in different cancer types, including gastric cancer and thyroid cancer.^40^^,^^50^^,^^51^ In our study, gene profiling, bioinformatics analysis and a series of *in vitro* and *in vivo* functional studies demonstrated that the SRCIN1/RASAL1-ERK signaling pathway largely accounts for the biological function of MEST in cancer invasion and metastasis, although other elements involved warrant further investigation. Our research strategy combining IP-MS and *in silico* transcription factor prediction successfully identified PURA as a key regulator in the mechanisms by which MEST affects SRCIN1 and RASAL1 expression. PURA is a ubiquitous multifunctional protein that is strongly conserved throughout evolution, binds to both DNA and RNA and functions in the initiation of DNA replication, transcriptional control and mRNA translation.^52^ The role of PURA in cancer, in particular, in transcriptional regulation, is complex and context dependent; for example, PURA can function as a transcriptional activator for some genes, including TGFβ1,^53^ TNFα,^54^ and β2-integrin.^55^ In contrast, a negative effect of PURA on the transcription of other genes, such as FAS and CD43, has also been reported.^56^^,^^57^ In this context, we illustrated the PURA-SRCIN1/RASAL1 regulatory axis as an important upstream mechanism of the ERK signaling pathway.

Emerging evidence suggests the important role of epigenetic abnormalities in the hallmarks of cancer.^58^^,^^59^ miRNAs can regulate gene expression by modulating the genome-wide epigenetic status of genes in various cancers.^60^ It was found that miR-335, which is harbored within an intron of its protein-coding host gene MEST, is downregulated by aberrant promoter hypermethylation in HCC. However, the exact mechanism by which miRNA regulates MEST to regulate ESCC progression remains unclear.^61^ Here, we report that miR-449a targets MEST to regulate SRCIN1/RASAL1-ERK-snail signaling and suppress ESCC metastasis, and deregulation of miR-449a in ESCC is associated with patient prognosis. The expression levels of miRNAs are coordinately modulated by different processes, such as methylation, RNA editing, and transcriptional regulation.^62^ DNA methylation, the process of adding a methyl group to cytosine to form 5-methylcytosine, can regulate gene expression by causing changes in chromatin structure, DNA conformation, DNA stability and DNA-protein interactions.^63^ In the present study, by integrating data from whole-genome methylation sequencing and methylation-specific PCR, our results revealed promoter methylation as an important mechanism for miR-449a downregulation in ESCC. In recent years, miRNAs have shown great potential for diagnostic and therapeutic applications in the treatment of human diseases. The first miRNA-based therapy approach in the treatment of cancer, MRX34, a miR-34a replacement, has also entered clinical testing.^64^ Our study showed that systemically delivered miR-449a mimic significantly inhibited ESCC metastasis in multiple experimental models, suggesting the potential of miR-449a as a key therapeutic agent against ESCC.

In addition to nucleic acid-based drugs and monoclonal antibodies, small-molecule drugs are still the mainstay of the pharmaceutical industry and have some distinct advantages as therapeutics, such as permeability, low cost, and oral administration. The rapid advancements in computational predictions and structure-based design and facilitate the development of small-molecule drugs. MEST is an attractive cancer target because it is upregulated in the majority of ESCC tumors examined, it is expressed at a low level in normal tissues, and its suppression has a minimal effect on normal cells. Our study integrated a combination of molecular docking, high-throughput invasion assay selection, SPR technology and protein–protein interaction inhibitor screening, leading to the discovery of lead compound G699-0288. A series of *in vitro* and *in vivo* functional assays demonstrated that by disturbing the MEST-PURA interaction, G699-0288 could suppress cancer invasion and metastasis without overt toxicity, suggesting the potential of developing G699-0288 as an anticancer agent against ESCC metastasis. In conclusion, we have identified the MEST-PURA-SRCIN1/RASAL1-ERK-snail signaling cascade as a key mechanism underlying cancer metastasis (Fig. 7l). The outcome of this study will facilitate the identification of prognostic biomarkers in ESCC, provide new mechanistic insight into the molecular pathogenesis of tumor metastasis and report useful preclinical data for the development of important systemic therapies for this lethal disease.

## Contributors

WWX, LL, WD, CCZ: acquisition and verification of data, analysis and interpretation of data, statistical analysis, drafting of the manuscript; XPT, YH, QHZ and ZHH: acquisition of data, analysis and interpretation of data; WYC, YRQ, KSC: technical and/or material support; MLH, SL: critical revision of the manuscript for important intellectual content; MLL, QYH, WWX, BL: funding acquisition, study concept and design, study supervision. These authors contributed equally: WWX, LL and WD. All authors reviewed and approved the final version of the manuscript.

## Data sharing statement

All data needed to evaluate the conclusions in the article are present in the article and/or the Supplementary Materials. The datasets used and/or analysed during the current study are available from the corresponding author on reasonable request. CRISPR/Cas9 screen NGS raw and preprocessed data is available in NCBI GEO (GSE211193).

## Declaration of interests

The authors have declared that no conflict of interest exists.

## Appendix ASupplementary data

Supplementary Figs. S1–S9

Supplemental Western blots
