# Supplementary material for: Genome-wide CRISPR/Cas9 screening identifies a targetable MEST-PURA interaction in cancer metastasis
Source: eBioMedicine. 2023 May 5;92:104587. doi: 10.1016/j.ebiom.2023.104587 (PMC10192437; doi:10.1016/j.ebiom.2023.104587)
Supplement: Supplementary Tables S3 [file mmc3.docx]

Table S3. The list of primers used for qRT-PCR.

| Primer name |  | Primer sequence |
| --- | --- | --- |
| BNC1 | Forward | 5'-AGTGGAGGGCTGTAATGCTACC-3' |
| BNC1 | Reverse | 5'-CTCTCCAATGCTTCCTGGCTCA-3' |
| ADRB2 | Forward | 5'-TACCAGAGCCTGCTGACCAAGA-3' |
| ADRB2 | Reverse | 5'-AGTCACAGCAGGTCTCATTGGC-3' |
| APLN | Forward | 5'-CCAGAGGGTCAAGGAATGGGC-3' |
| APLN | Reverse | 5'-ATAACCGCCGGGGGTGGGCA-3' |
| RASAL1 | Forward | 5'-CTCATGGAGTCTGTGCAGGG-3' |
| RASAL1 | Reverse | 5'-CCCAAGGTCAGCTCTTCCAG-3' |
| SRCIN1 | Forward | 5'-CATGGTGCTGGTGAAAGGC-3' |
| SRCIN1 | Reverse | 5'-GAGCCGCGCTTGTAGAGG-3' |
