# Supplementary material for: Genome-wide CRISPR/Cas9 screening identifies a targetable MEST-PURA interaction in cancer metastasis
Source: eBioMedicine. 2023 May 5;92:104587. doi: 10.1016/j.ebiom.2023.104587 (PMC10192437; doi:10.1016/j.ebiom.2023.104587)
Supplement: Supplementary Tables S4 [file mmc4.docx]

| Variable | n | Low SRCIN1 | High SRCIN1 | *P* value |
| --- | --- | --- | --- | --- |
| Age (years) |  |  |  |  |
| ≤55 | 47 | 37 | 10 |  |
| >55 | 195 | 145 | 50 | 0.533 |
|  |  |  |  |  |
| Gender |  |  |  |  |
| Female | 58 | 44 | 14 |  |
| Male | 184 | 138 | 46 | 1.000 |
|  |  |  |  |  |
| T-Stage |  |  |  |  |
| 1/2 | 46 | 33 | 13 |  |
| 3/4 | 179 | 134 | 45 | 0.665 |
|  |  |  |  |  |
| N-Stage |  |  |  |  |
| N0 | 115 | 80 | 35 |  |
| N1/N2/N3 | 124 | 100 | 24 | **0.047*** |
|  |  |  |  |  |
| M-Stage |  |  |  |  |
| M0 | 237 | 178 | 59 |  |
| M1 | 4 | 3 | 1 | 1.000 |
|  |  |  |  |  |
| Grade |  |  |  |  |
| I & II | 187 | 138 | 49 |  |
| III & IV | 55 | 44 | 11 | 0.349 |
|  |  |  |  |  |
|  |  |  |  |  |

Table S4. Correlation between SRCIN1 expression levels and clinicopathological parameters in 242 cases of esophageal cancer.
