# Supplementary material for: Genome-wide CRISPR/Cas9 screening identifies a targetable MEST-PURA interaction in cancer metastasis
Source: eBioMedicine. 2023 May 5;92:104587. doi: 10.1016/j.ebiom.2023.104587 (PMC10192437; doi:10.1016/j.ebiom.2023.104587)
Supplement: Supplementary Tables S5 [file mmc5.docx]

| Variable | n | Low RASAL1 | High RASAL1 | *P* value |
| --- | --- | --- | --- | --- |
| Age (years) |  |  |  |  |
| ≤55 | 47 | 34 | 13 |  |
| >55 | 195 | 156 | 39 | p=0.251 |
|  |  |  |  |  |
| Gender |  |  |  |  |
| Female | 58 | 46 | 12 |  |
| Male | 184 | 144 | 40 | p=0.865 |
|  |  |  |  |  |
| T-Stage |  |  |  |  |
| 1/2 | 46 | 31 | 15 |  |
| 3/4 | 179 | 147 | 32 | **p=0.028*** |
|  |  |  |  |  |
| N-Stage |  |  |  |  |
| N0 | 116 | 85 | 31 |  |
| N1/N2/N3 | 124 | 105 | 19 |  |
|  |  |  |  | **p=0.029*** |
| M-Stage |  |  |  |  |
| M0 | 237 | 186 | 51 |  |
| M1 | 4 | 4 | 0 | p=0.16 |
|  |  |  |  |  |
| Grade |  |  |  |  |
| I & II | 187 | 144 | 43 |  |
| III & IV | 55 | 46 | 9 | p=0.292 |
|  |  |  |  |  |
|  |  |  |  |  |

Table S5. Correlation between RASAL1 expression levels and clinicopathological parameters in 242 cases of esophageal cancer.
