# Supplementary material for: Genome-wide CRISPR/Cas9 screening identifies a targetable MEST-PURA interaction in cancer metastasis
Source: eBioMedicine. 2023 May 5;92:104587. doi: 10.1016/j.ebiom.2023.104587 (PMC10192437; doi:10.1016/j.ebiom.2023.104587)
Supplement: Supplementary Tables S6 [file mmc6.docx]

Table S6. The interacting proteins was identified from immunoprecipitation coupled with liquid chromatography tandem mass spectrometry (IP-MS)

|  | Entry | Protein names | Gene ID |
| --- | --- | --- | --- |
| 1 | Q9Y6Y0 | Influenza virus NS1A-binding protein (NS1-BP) (NS1-binding protein) (Aryl hydrocarbon receptor-associated protein 3) | IVNS1ABP ARA3 FLARA3 KIAA0850 NS1 NS1BP HSPC068 |
| 2 | Q9BVI4 | Nucleolar complex protein 4 homolog (NOC4 protein homolog) (NOC4-like protein) (Nucleolar complex-associated protein 4-like protein) | NOC4L |
| 3 | P78316 | Nucleolar protein 14 (Nucleolar complex protein 14) | NOP14 C4orf9 NOL14 RES4-25 |
| 4 | Q13310 | Polyadenylate-binding protein 4 (PABP-4) (Poly(A)-binding protein 4) (Activated-platelet protein 1) (APP-1) (Inducible poly(A)-binding protein) (iPABP) | PABPC4 APP1 PABP4 |
| 5 | Q9NZB2 | Constitutive coactivator of PPAR-gamma-like protein 1 (Oxidative stress-associated Src activator) (Protein FAM120A) | FAM120A C9orf10 KIAA0183 OSSA |
| 6 | Q8IWX8 | Calcium homeostasis endoplasmic reticulum protein (ERPROT 213-21) (SR-related CTD-associated factor 6) | CHERP DAN26 SCAF6 |
| 7 | Q9BZF1 | Oxysterol-binding protein-related protein 8 (ORP-8) (OSBP-related protein 8) | OSBPL8 KIAA1451 ORP8 OSBP10 |
| 8 | P62263 | 40S ribosomal protein S14 (Small ribosomal subunit protein uS11) | RPS14 PRO2640 |
| 9 | P07996 | Thrombospondin-1 | THBS1 TSP TSP1 |
| 10 | Q9NWU2 | Glucose-induced degradation protein 8 homolog (Two hybrid-associated protein 1 with RanBPM) (Twa1) | GID8 C20orf11 |
| 11 | P62314 | Small nuclear ribonucleoprotein Sm D1 (Sm-D1) (Sm-D autoantigen) (snRNP core protein D1) | SNRPD1 |
| 12 | Q03135 | Caveolin-1 | CAV1 CAV |
| 13 | P09012 | U1 small nuclear ribonucleoprotein A (U1 snRNP A) (U1-A) (U1A) | SNRPA |
| 14 | P60866 | 40S ribosomal protein S20 (Small ribosomal subunit protein uS10) | RPS20 |
| 15 | P62316 | Small nuclear ribonucleoprotein Sm D2 (Sm-D2) (snRNP core protein D2) | SNRPD2 SNRPD1 |
| 16 | Q00577 | Transcriptional activator protein Pur-alpha (Purine-rich single-stranded DNA-binding protein alpha) | PURA PUR1 |
| 17 | Q96I25 | Splicing factor 45 (45 kDa-splicing factor) (RNA-binding motif protein 17) | RBM17 SPF45 |
| 18 | O15479 | Melanoma-associated antigen B2 (Cancer/testis antigen 3.2) (CT3.2) (DSS-AHC critical interval MAGE superfamily 6) (DAM6) (MAGE XP-2 antigen) (MAGE-B2 antigen) | MAGEB2 |
| 19 | P62318 | Small nuclear ribonucleoprotein Sm D3 (Sm-D3) (snRNP core protein D3) | SNRPD3 |
| 20 | Q9NSB2 | Keratin, type II cuticular Hb4 (Keratin-84) (K84) (Type II hair keratin Hb4) (Type-II keratin Kb24) | KRT84 KRTHB4 |
| 21 | Q92896 | Golgi apparatus protein 1 (CFR-1) (Cysteine-rich fibroblast growth factor receptor) (E-selectin ligand 1) (ESL-1) (Golgi sialoglycoprotein MG-160) | GLG1 CFR1 ESL1 MG160 |
| 22 | P51398 | 28S ribosomal protein S29, mitochondrial (MRP-S29) (S29mt) (Death-associated protein 3) (DAP-3) (Ionizing radiation resistance conferring protein) (Mitochondrial small ribosomal subunit protein mS29) | DAP3 MRPS29 |
| 23 | P49902 | Cytosolic purine 5'-nucleotidase (EC 3.1.3.5) (Cytosolic 5'-nucleotidase II) | NT5C2 NT5B NT5CP PNT5 |
| 24 | P62888 | 60S ribosomal protein L30 (Large ribosomal subunit protein eL30) | RPL30 |
| 25 | P62249 | 40S ribosomal protein S16 (Small ribosomal subunit protein uS9) | RPS16 |
| 26 | Q13509 | Tubulin beta-3 chain (Tubulin beta-4 chain) (Tubulin beta-III) | TUBB3 TUBB4 |
| 27 | P22626 | Heterogeneous nuclear ribonucleoproteins A2/B1 (hnRNP A2/B1) | HNRNPA2B1 HNRPA2B1 |
| 28 | P78362 | SRSF protein kinase 2 (EC 2.7.11.1) (SFRS protein kinase 2) (Serine/arginine-rich protein-specific kinase 2) (SR-protein-specific kinase 2) [Cleaved into: SRSF protein kinase 2 N-terminal; SRSF protein kinase 2 C-terminal] | SRPK2 |
| 29 | Q92900 | Regulator of nonsense transcripts 1 (EC 3.6.4.-) (ATP-dependent helicase RENT1) (Nonsense mRNA reducing factor 1) (NORF1) (Up-frameshift suppressor 1 homolog) (hUpf1) | UPF1 KIAA0221 RENT1 |
| 30 | P14678 | Small nuclear ribonucleoprotein-associated proteins B and B' (snRNP-B) (Sm protein B/B') (Sm-B/B') (SmB/B') | SNRPB COD SNRPB1 |
| 31 | P57721 | Poly(rC)-binding protein 3 (Alpha-CP3) (PCBP3-overlapping transcript) (PCBP3-overlapping transcript 1) | PCBP3 PCBP3-OT1 PCBP3OT |
| 32 | P51991 | Heterogeneous nuclear ribonucleoprotein A3 (hnRNP A3) | HNRNPA3 HNRPA3 |
| 33 | Q96G75 | Protein RMD5 homolog B | RMND5B UNQ2508/PRO5996 |
| 34 | Q15427 | Splicing factor 3B subunit 4 (Pre-mRNA-splicing factor SF3b 49 kDa subunit) (Spliceosome-associated protein 49) (SAP 49) | SF3B4 SAP49 |
| 35 | P25398 | 40S ribosomal protein S12 (Small ribosomal subunit protein eS12) | RPS12 |
| 36 | P22087 | rRNA 2'-O-methyltransferase fibrillarin (EC 2.1.1.-) (34 kDa nucleolar scleroderma antigen) (Histone-glutamine methyltransferase) | FBL FIB1 FLRN |
| 37 | P37108 | Signal recognition particle 14 kDa protein (SRP14) (18 kDa Alu RNA-binding protein) | SRP14 |
| 38 | Q9H501 | ESF1 homolog (ABT1-associated protein) | ESF1 ABTAP C20orf6 HDCMC28P |
| 39 | P33992 | DNA replication licensing factor MCM5 (EC 3.6.4.12) (CDC46 homolog) (P1-CDC46) | MCM5 CDC46 |
| 40 | Q92499 | ATP-dependent RNA helicase DDX1 (EC 3.6.4.13) (DEAD box protein 1) (DEAD box protein retinoblastoma) (DBP-RB) | DDX1 |
| 41 | Q16637 | Survival motor neuron protein (Component of gems 1) (Gemin-1) | SMN1 SMN SMNT; SMN2 SMNC |
| 42 | Q6ZUT6 | Uncharacterized protein C15orf52 | C15orf52 |
| 43 | Q9BZH6 | WD repeat-containing protein 11 (Bromodomain and WD repeat-containing protein 2) (WD repeat-containing protein 15) | WDR11 BRWD2 KIAA1351 WDR15 |
| 44 | P46060 | Ran GTPase-activating protein 1 (RanGAP1) | RANGAP1 KIAA1835 SD |
| 45 | P51636 | Caveolin-2 | CAV2 |
| 46 | P62244 | 40S ribosomal protein S15a (Small ribosomal subunit protein uS8) | RPS15A OK/SW-cl.82 |
| 47 | Q9Y3X0 | Coiled-coil domain-containing protein 9 | CCDC9 |
| 48 | Q92804 | TATA-binding protein-associated factor 2N (68 kDa TATA-binding protein-associated factor) (TAF(II)68) (TAFII68) (RNA-binding protein 56) | TAF15 RBP56 TAF2N |
| 49 | Q7Z7A4 | PX domain-containing protein kinase-like protein (Modulator of Na,K-ATPase) (MONaKA) | PXK |
| 50 | Q9Y383 | Putative RNA-binding protein Luc7-like 2 | LUC7L2 CGI-59 CGI-74 |
| 51 | Q9UJF2 | Ras GTPase-activating protein nGAP (RAS protein activator-like 2) | RASAL2 NGAP |
| 52 | Q8WVF1 | Protein OSCP1 (hOSCP1) (Organic solute transport protein 1) (Oxidored-nitro domain-containing protein 1) | OSCP1 C1orf102 NOR1 |
| 53 | O95218 | Zinc finger Ran-binding domain-containing protein 2 (Zinc finger protein 265) (Zinc finger, splicing) | ZRANB2 ZIS ZNF265 |
| 54 | P61129 | Zinc finger CCCH domain-containing protein 6 | ZC3H6 KIAA2035 ZC3HDC6 |
| 55 | Q6PKG0 | La-related protein 1 (La ribonucleoprotein domain family member 1) | LARP1 KIAA0731 LARP |
| 56 | Q15643 | Thyroid receptor-interacting protein 11 (TR-interacting protein 11) (TRIP-11) (Clonal evolution-related gene on chromosome 14 protein) (Golgi-associated microtubule-binding protein 210) (GMAP-210) (Trip230) | TRIP11 CEV14 |
| 57 | Q02413 | Desmoglein-1 (Cadherin family member 4) (Desmosomal glycoprotein 1) (DG1) (DGI) (Pemphigus foliaceus antigen) | DSG1 CDHF4 |
| 58 | O14980 | Exportin-1 (Exp1) (Chromosome region maintenance 1 protein homolog) | XPO1 CRM1 |
| 59 | Q8WXD5 | Gem-associated protein 6 (Gemin-6) (SIP2) | GEMIN6 |
| 60 | Q9UPM8 | AP-4 complex subunit epsilon-1 (AP-4 adaptor complex subunit epsilon) (Adaptor-related protein complex 4 subunit epsilon-1) (Epsilon subunit of AP-4) (Epsilon-adaptin) | AP4E1 |
| 61 | Q8TEA8 | D-aminoacyl-tRNA deacylase 1 (DTD) (EC 3.1.1.96) (DNA-unwinding element-binding protein B) (DUE-B) (Gly-tRNA(Ala) deacylase) (EC 3.1.1.-) (Histidyl-tRNA synthase-related) | DTD1 C20orf88 DUEB HARS2 |
| 62 | O95602 | DNA-directed RNA polymerase I subunit RPA1 (RNA polymerase I subunit A1) (EC 2.7.7.6) (A190) (DNA-directed RNA polymerase I largest subunit) (DNA-directed RNA polymerase I subunit A) (RNA polymerase I 194 kDa subunit) (RPA194) | POLR1A |
