# Supplementary material for: Genome-wide CRISPR/Cas9 screening identifies a targetable MEST-PURA interaction in cancer metastasis
Source: eBioMedicine. 2023 May 5;92:104587. doi: 10.1016/j.ebiom.2023.104587 (PMC10192437; doi:10.1016/j.ebiom.2023.104587)
Supplement: Supplementary Tables S7 [file mmc7.docx]

Table S7. Primer lists for generating mutant of SRCINI1 and RASAL1 promoter plasmids.

| Primer ID |  | Sequence |
| --- | --- | --- |
| RASAL1-BS1-M | Forward | 5'-GGGGAGGGGGGTGGAGGGGTGGGGG-3' |
|  | Reverse | 5'-CCCCCACCCCTCCACCCCCCTCCCC-3' |
| SRCIN1-BS4-M | Forward | 5'-GGGGCGGGGGTCGGGAGGGTGGAGC-3' |
|  | Reverse | 5'-GCTCCACCCTCCCGACCCCCGCCCC-3' |
| SRCIN1-BS5-M | Forward | 5'-GCTTCCCCGGGAGCCACCTCCGAGCCG-3' |
|  | Reverse | 5'-CGGCTCGGAGGTGGCTCCCGGGGAAGC-3' |
