# Supplementary material for: Genome-wide CRISPR/Cas9 screening identifies a targetable MEST-PURA interaction in cancer metastasis
Source: eBioMedicine. 2023 May 5;92:104587. doi: 10.1016/j.ebiom.2023.104587 (PMC10192437; doi:10.1016/j.ebiom.2023.104587)
Supplement: Supplementary Tables S8 [file mmc8.docx]

Table S8. The sequences of PURA-targeting and MEST-targeting sgRNA.

| Primer name | Primer sequence |
| --- | --- |
| PURA-sgRNA | 5'-ATCATGGCGGACCGAGACAG-3' |
| MEST-sgRNA | 5'-TTTCCTACAGGATGAGGGAG-3' |
