# Supplementary material for: Genome-wide CRISPR/Cas9 screening identifies a targetable MEST-PURA interaction in cancer metastasis
Source: eBioMedicine. 2023 May 5;92:104587. doi: 10.1016/j.ebiom.2023.104587 (PMC10192437; doi:10.1016/j.ebiom.2023.104587)
Supplement: Supplementary Tables S9 [file mmc9.docx]

| Variable | n | Low miR-449 | High miR-449 | *P* value |
| --- | --- | --- | --- | --- |
| Age (years) |  |  |  |  |
| ≤55 | 47 | 37 | 10 |  |
| >55 | 195 | 133 | 62 | 0.156 |
|  |  |  |  |  |
| Gender |  |  |  |  |
| Female | 58 | 39 | 19 |  |
| Male | 184 | 131 | 53 | 0.565 |
|  |  |  |  |  |
| T-Stage |  |  |  |  |
| 1/2 | 46 | 25 | 21 |  |
| 3/4 | 179 | 133 | 46 | **0.008**** |
|  |  |  |  |  |
| N-Stage |  |  |  |  |
| N0 | 115 | 64 | 51 |  |
| N1/N2/N3 | 124 | 104 | 21 | **0.001***** |
|  |  |  |  |  |
| M-Stage |  |  |  |  |
| M0 | 238 | 166 | 72 |  |
| M1 | 4 | 4 | 0 | 0.087 |
|  |  |  |  |  |
| Grade |  |  |  |  |
| I & II | 187 | 128 | 59 |  |
| III & IV | 55 | 42 | 13 | 0.259 |
|  |  |  |  |  |
|  |  |  |  |  |

Table S9. Correlation between miR-449 expression levels and clinicopathological parameters in 242 cases of esophageal cancer.
