# Supplementary material for: Genome-wide CRISPR/Cas9 screening identifies a targetable MEST-PURA interaction in cancer metastasis
Source: eBioMedicine. 2023 May 5;92:104587. doi: 10.1016/j.ebiom.2023.104587 (PMC10192437; doi:10.1016/j.ebiom.2023.104587)
Supplement: Supplementary Tables S11 [file mmc11.docx]

Table S11. The methylated or unmethylated primers for methylation specific-PCR (MSP) analysis.

| Primer name |  | Primer sequence |
| --- | --- | --- |
| methylated | Forward | 5'-TTTATTTGAAATGTTGTATTGG-3' |
|  | Reverse | 5'-TTCATAAAAACTATATAACTATAATAACGA-3' |
| unmethylated | Forward | 5'-TTTATTTGAAATGTTGTATTGG-3' |
|  | Reverse | 5'-ATTCATAAAAACTATATAACTATAATAACA-3' |
