# Supplementary material for: Genome-wide CRISPR/Cas9 screening identifies a targetable MEST-PURA interaction in cancer metastasis
Source: eBioMedicine. 2023 May 5;92:104587. doi: 10.1016/j.ebiom.2023.104587 (PMC10192437; doi:10.1016/j.ebiom.2023.104587)
Supplement: Supplementary Tables S12 [file mmc12.docx]

Table S12. Primer lists for generating MEST 3’UTR-mutant plasmids.

| Primer name |  | Primer sequence |
| --- | --- | --- |
| MEST 3’UTR | Forward | 5'-CCTCTCTGGGACAGCCTGTGGGGAGTTAAGC-3' |
| MEST 3’UTR | Reverse | 5'-GCTTAACTCCCCACAGGCTGTCCCAGAGAGG-3' |
