# Supplementary material for: Genome-wide CRISPR/Cas9 screening identifies a targetable MEST-PURA interaction in cancer metastasis
Source: eBioMedicine. 2023 May 5;92:104587. doi: 10.1016/j.ebiom.2023.104587 (PMC10192437; doi:10.1016/j.ebiom.2023.104587)
Supplement: Supplementary Figs. S1–S9 [file mmc14.pdf]

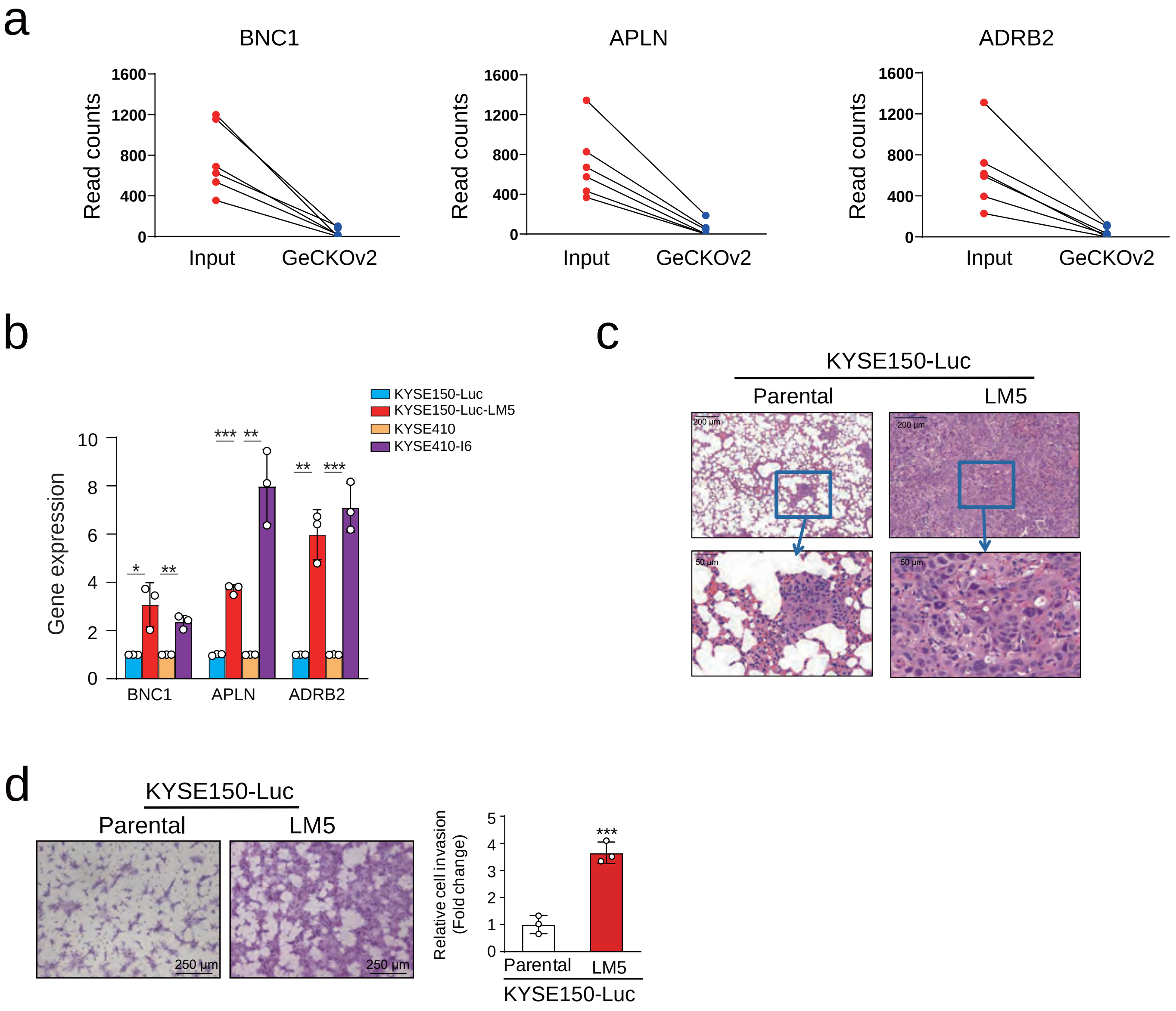

a
BNC1
APLN
ADRB2
Read counts
1600
1200
800
400
0
Input
GeCKOv2
b
KYSE150-Luc
KYSE150-Luc-LM5
KYSE410
KYSE410-I6
Gene expression
10
8
6
4
2
0
*
**
***
BNC1
APLN
ADRB2
c
KYSE150-Luc
Parental
LM5
d
KYSE150-Luc
Parental
LM5
250 μm
Relative cell invasion (Fold change)
5
4
3
2
1
0
***
Parental
LM5
KYSE150-Luc

Figure S1

Supplementary Fig 1. (a) Comparison of read counts of individual sgRNAs targeting BNC1, APLN and ADRB2 in GeCKOv2 and input cells. **(b)** The mRNA expression levels of BNC1, APLN and ADRB2 in KYSE150-Luc-LM5 and KYSE410-I6 cells compared with their parental cells. **(c)** Histological analysis of lungs from the mice intravenously injected with KYSE150-Luc and KYSE150-Luc-LM5 cells (n=3). **(d)** Boyden chamber assay comparing the invasion of KYSE150-Luc-LM5 and parental cells. Bars, SD; *, $P < 0.05$; **, $P < 0.01$; ***, $P < 0.001$, the student's *t* test.

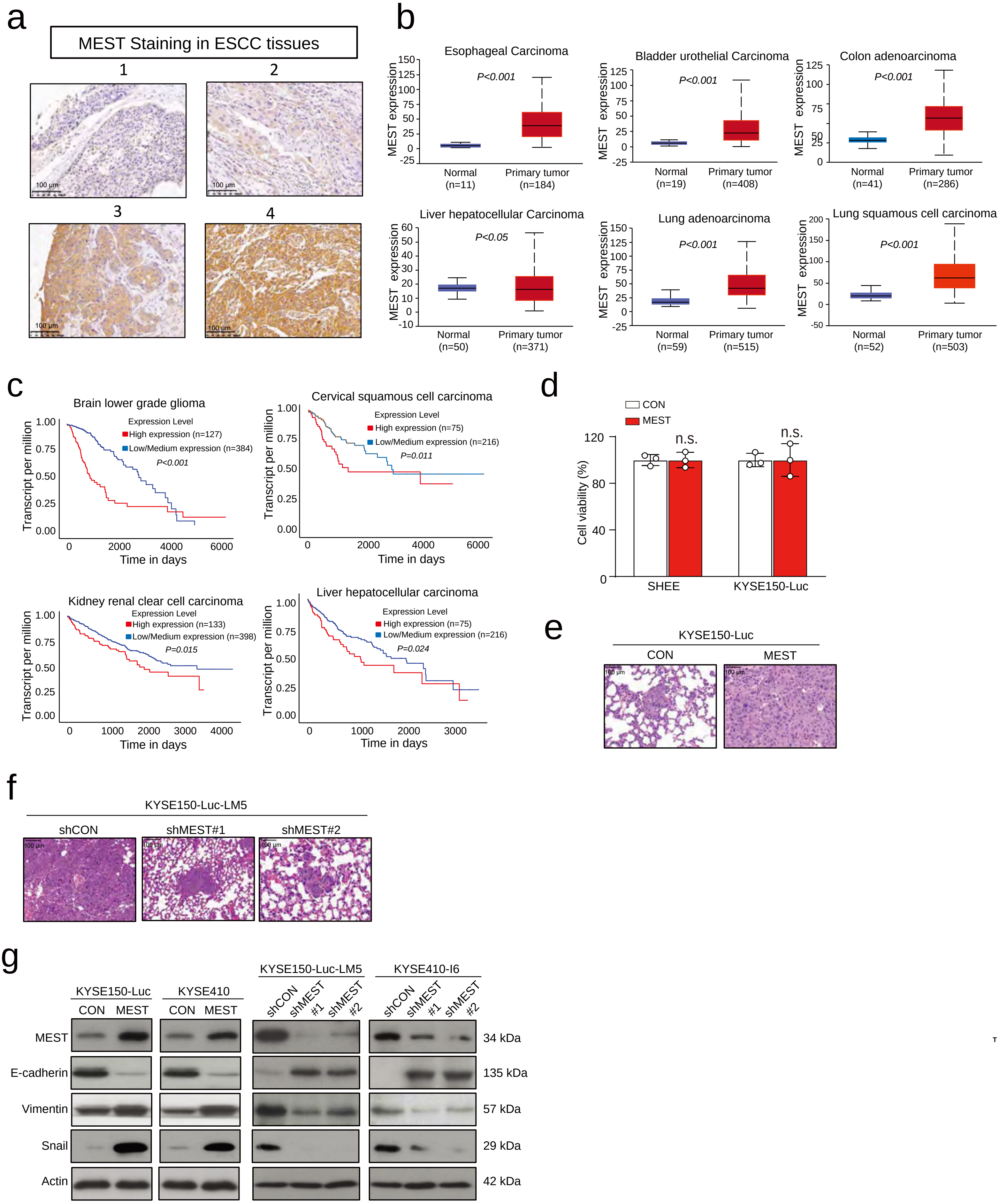
a
MEST Staining in ESCC tissues
1
2
3
4
b
Esophageal Carcinoma
Bladder urothelial Carcinoma
Colon adenoarcinoma
Liver hepatocellular Carcinoma
Lung adenoarcinoma
Lung squamous cell carcinoma
MEST expression
Normal
Primary tumor
c
Brain lower grade glioma
Cervical squamous cell carcinoma
Kidney renal clear cell carcinoma
Liver hepatocellular carcinoma
Expression Level
Transcript per million
Time in days
d
CON
MEST
Cell viability (%)
n.s.
SHEE
KYSE150-Luc
e
KYSE150-Luc
CON
MEST
f
KYSE150-Luc-LM5
shCON
shMEST#1
shMEST#2
g
KYSE150-Luc
KYSE410
KYSE150-Luc-LM5
KYSE410-I6
MEST
E-cadherin
Vimentin
Snail
Actin
34 kDa
135 kDa
57 kDa
29 kDa
42 kDa

Figure S2

Supplementary Fig. 2. (a) Representative images of the different scores for MEST in ESCC. **(b)** MEST expression in different cancer types in the TCGA database is upregulated in tumor tissues compared with normal tissues. **(c)** Kaplan-Meier analysis showing the expression of MEST and patient survival in different cancers. **(d)** CCK8 assay showing MEST had on effect on proliferation of esophageal cell line SHEE and esophageal cancer cell line KYSE150. **(e-f)** H&E staining showing the lungs from the mice intravenously injected with the indicated cell lines (n=3). **(g)** Western Blot showing the effect of manipulation of MEST expression on EMT markers, including E-cadherin, Vimentin, Snail. Bars, SD; **, $P < 0.01$; ***, $P < 0.001$, the student's *t* test.

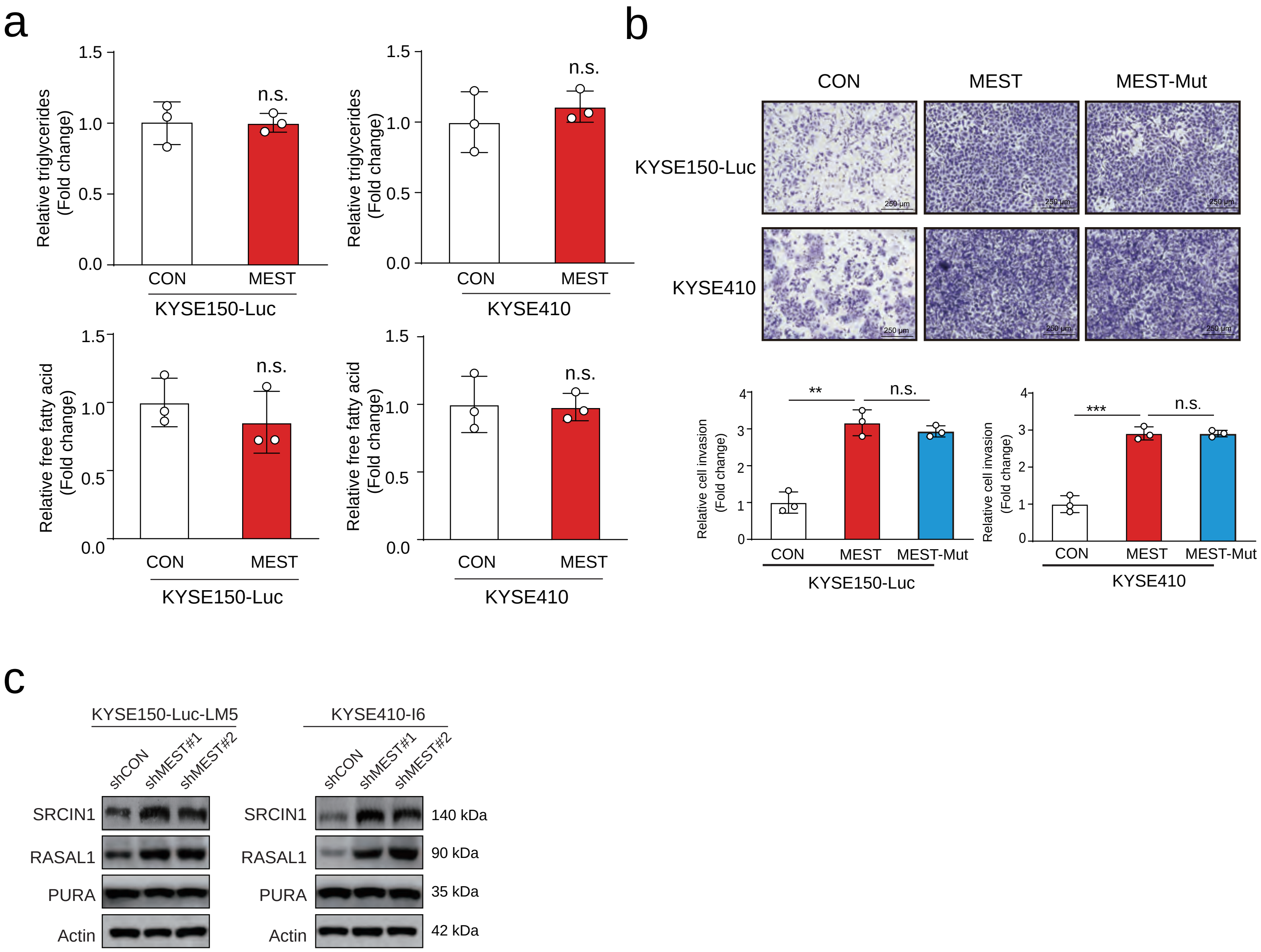
a
Relative triglycerides (Fold change)
1.5
1.0
0.5
0.0
n.s.
CON
MEST
KYSE150-Luc
KYSE410
Relative free fatty acid (Fold change)
b
CON
MEST
MEST-Mut
KYSE150-Luc
KYSE410
250 μm
Relative cell invasion (Fold change)
**
***
c
KYSE150-Luc-LM5
KYSE410-I6
shCON
shMEST#1
shMEST#2
SRCIN1
RASAL1
PURA
Actin
140 kDa
90 kDa
35 kDa
42 kDa

Figure S3

Supplementary Fig. 3. (a) The levels of free fatty acids and triglyceride were determined in the MEST-overexpressing ESCC cells. **(b)** Mutation of the hydrolase site of MEST did not affect the role of MEST in regulating ESCC cell invasion. **(c)** The protein expression levels of SRCIN1 and RASAL1 in MEST-knockdown KYSE150-Luc-LM5 and KYSE410-I6 cells. Bars, SD; ***, $P < 0.001$, the student's *t* test.

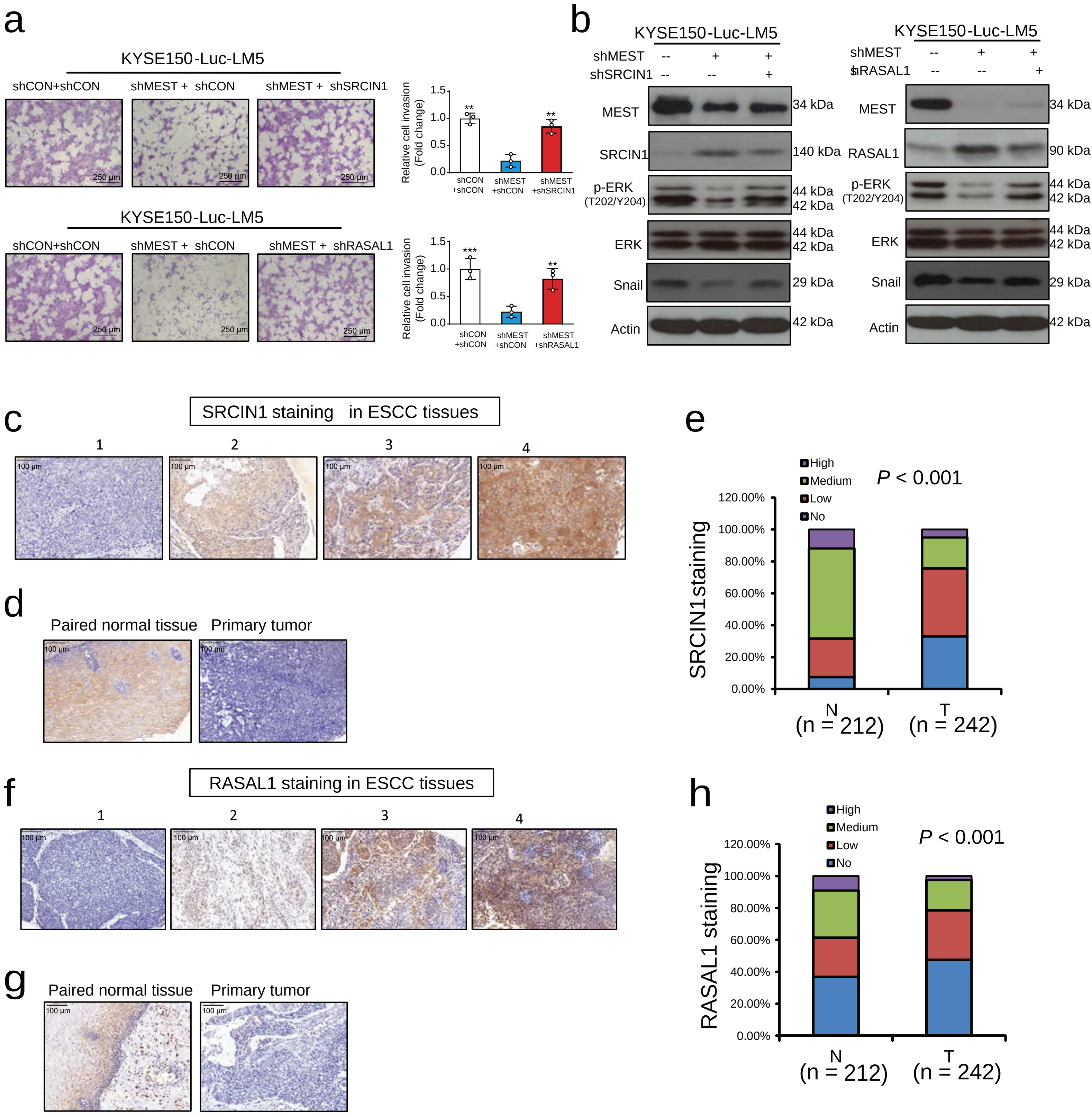

a
KYSE150-Luc-LM5
shCON+shCON
shMEST + shCON
shMEST + shSRCIN1
shMEST + shRASAL1
Relative cell invasion (Fold change)
shCON +shCON
shMEST +shCON
shMEST +shSRCIN1
shMEST +shRASAL1
b
shMEST
shSRCIN1
shRASAL1
MEST
SRCIN1
RASAL1
p-ERK (T202/Y204)
ERK
Snail
Actin
34 kDa
140 kDa
90 kDa
44 kDa
42 kDa
29 kDa
c
SRCIN1 staining in ESCC tissues
d
Paired normal tissue
Primary tumor
e
SRCIN1staining
High
Medium
Low
No
P < 0.001
N (n = 212)
T (n = 242)
f
RASAL1 staining in ESCC tissues
g
h
RASAL1 staining

Figure S4

Supplementary Fig. 4. (a) SRCIN1 and RASAL1 were further silenced in MEST-knockdown ESCC cells, and a Boyden chamber assay was performed to determine the cell invasion ability. **(b)** Western blot showing the expression of p-ERK, ERK and snail in MEST-knockdown ESCC cells when SRCIN1 and RASAL1 were further knocked down. **(c)** Representative images of the different scores for SRCIN1 in ESCC. **(d)** Representative images of SRCIN1 in primary tumor and paired normal tissues. **(e)** Expression pattern of SRCIN1 in 242 ESCC tumor and 212 paired normal tissues. **(f)** Representative images of the different scores for RASAL1 in ESCC. **(g)** Representative images of RASAL1 in primary tumor and paired normal tissue. **(h)** Expression pattern of RASAL1 in 242 ESCC tumor and 212 paired normal tissues. Bars, SD; **, $P < 0.01$; ***, $P < 0.001$, the student's *t* test.

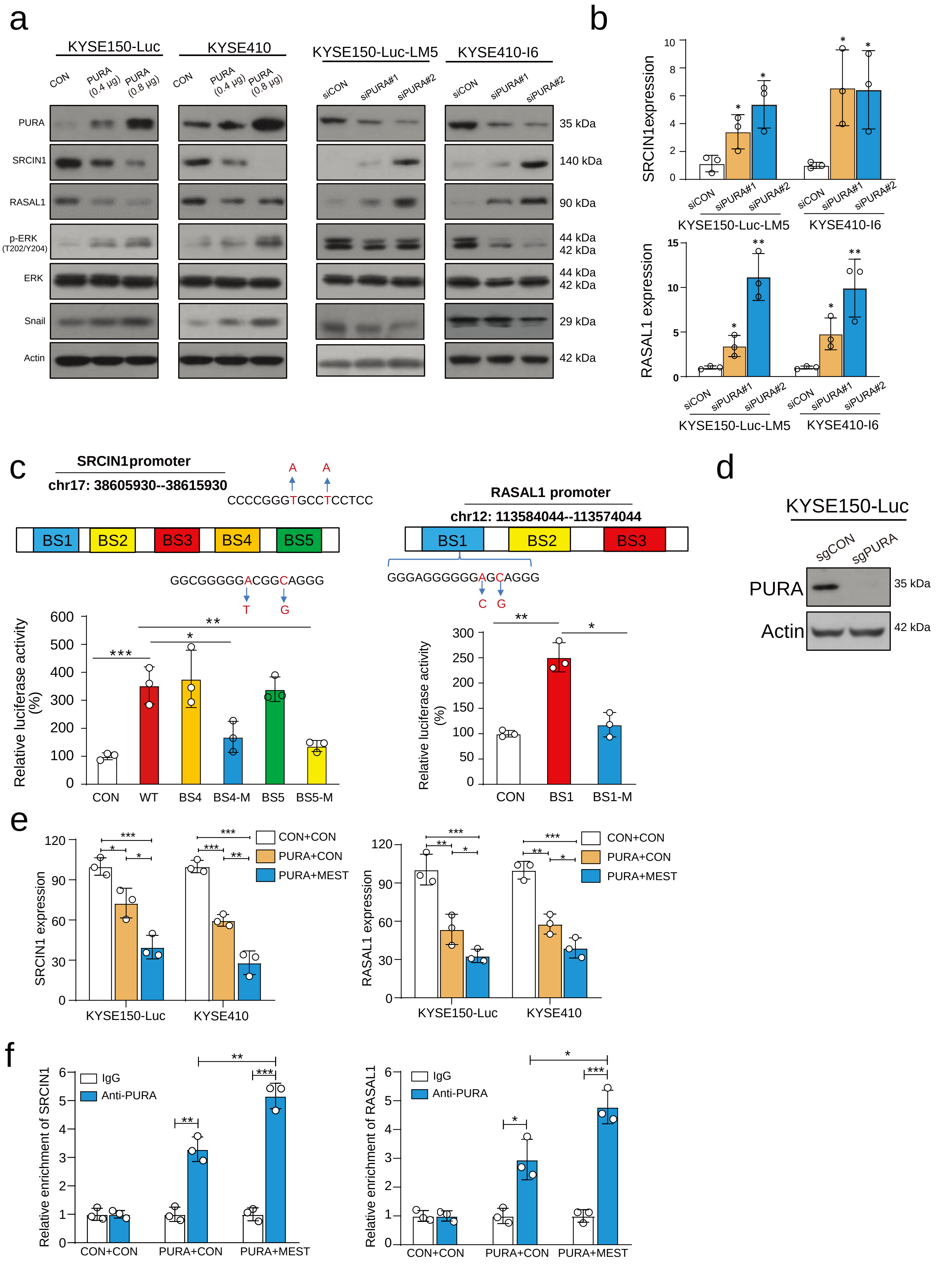
a
KYSE150-Luc
KYSE410
CON
PURA (0.4 μg)
PURA (0.8 μg)
KYSE150-Luc-LM5
KYSE410-I6
siCON
siPURA#1
siPURA#2
PURA
SRCIN1
RASAL1
p-ERK (T202/Y204)
ERK
Snail
Actin
35 kDa
140 kDa
90 kDa
44 kDa
42 kDa
29 kDa
b
SRCIN1expression
RASAL1 expression
c
SRCIN1promoter
chr17: 38605930--38615930
CCCCGGGTGCCTCCTCC
BS1
BS2
BS3
BS4
BS5
GGCGGGGGACGGCAGGG
RASAL1 promoter
chr12: 113584044--113574044
GGGAGGGGGGAGCAGGG
Relative luciferase activity (%)
CON
WT
BS4-M
BS5-M
BS1-M
d
sgCON
sgPURA
e
SRCIN1 expression
RASAL1 expression
CON+CON
PURA+CON
PURA+MEST
f
Relative enrichment of SRCIN1
Relative enrichment of RASAL1
IgG
Anti-PURA

Figure S5

Supplementary Fig. 5. (a) ESCC cells were transfected with different concentrations of PURA overexpression plasmid or siRNA against PURA, and Western blotting was used to detect the expression of SRCIN1, RASAL1, p-ERK and ERK. **(b)** The qRT-PCR data showing the effect of PURA-knockdown on the mRNA expression levels of SRCIN1 and RASAL1 in KYSE150-Luc-LM5 and KYSE410-I6 cells. **(c)** Diagram illustrating the site-specific mutations introduced in the reporter plasmids (upper panel). The luciferase activity in cells transfected with the wild type or mutated promoters of SRCIN1 and RASAL1 was determined in the presence or absence of PURA overexpression. **(d)** Establishment of the PURA-knockout cell line by the CRISPR/Cas9 system. **(e)** The qRT-PCR data showing MEST increased the effect of PURA on the mRNA expression levels of SRCIN1 and RASAL1 in ESCC cells. **(f)** ChIP assay showing MEST increased the enrichment of PURA in the promoter regions of SRCIN1 and RASAL1. Bars, SD; *, $P < 0.05$; **, $P < 0.01$; ***, $P < 0.001$, the student's *t* test.

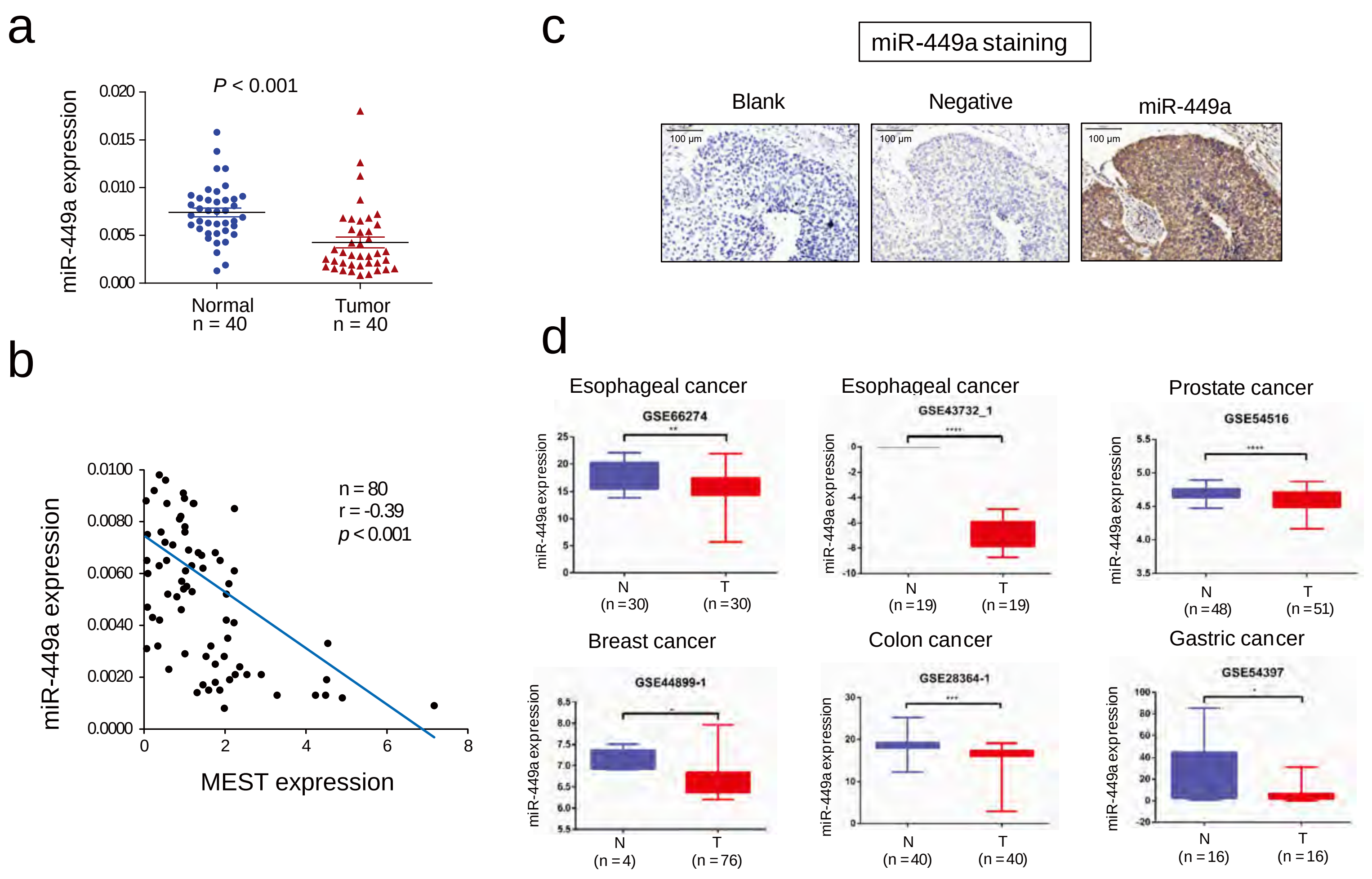
a
P < 0.001
miR-449a expression
0.020
0.015
0.010
0.005
0.000
Normal
n = 40
Tumor
n = 40
b
miR-449a expression
0.0100
0.0080
0.0060
0.0040
0.0020
0.0000
0
2
4
6
8
MEST expression
n = 80
r = -0.39
p < 0.001
c
miR-449a staining
Blank
Negative
miR-449a
100 μm
d
Esophageal cancer
GSE66274
Esophageal cancer
GSE43732_1
Prostate cancer
GSE54516
Breast cancer
GSE44899-1
Colon cancer
GSE28364-1
Gastric cancer
GSE54397
miR-449a expression
N
T
(n = 30)
(n = 30)
(n = 19)
(n = 19)
(n = 48)
(n = 51)
(n = 4)
(n = 76)
(n = 40)
(n = 40)
(n = 16)
(n = 16)

Figure S6

Supplementary Fig. 6. (a) TaqMan qPCR assay showing miR-449a expression in 40 ESCC tumor and paired normal tissues. **(b)** Association between MEST and miR-449a expression in ESCC. **(c)** Representative images of the different scores for miR-449a in ESCC. **(d)** The expression and clinical relevance of miR-449a in public databases. Bars, SD; *, $P < 0.05$; **, $P < 0.01$; ***, $P < 0.001$, the student's t test.

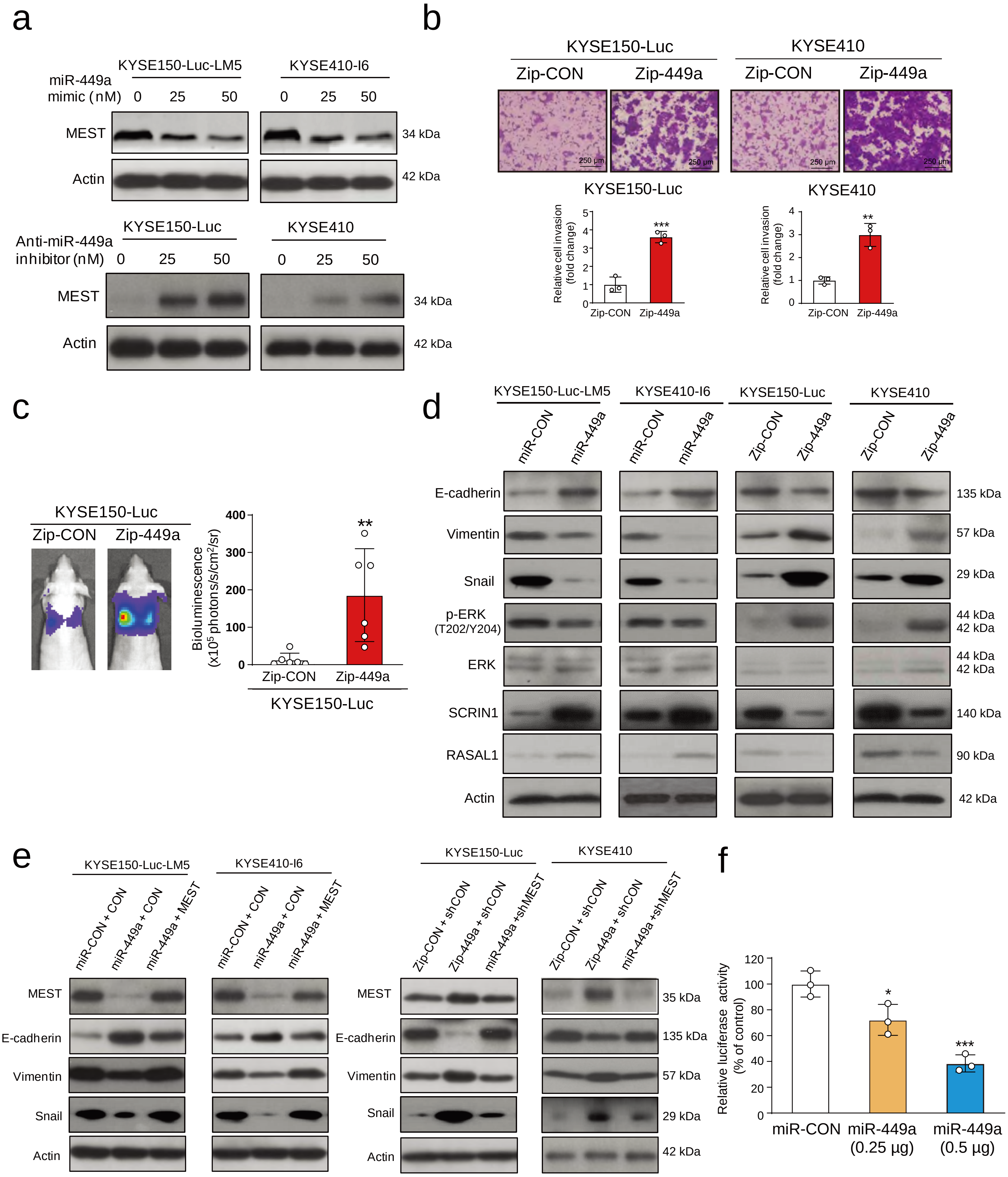
a
KYSE150-Luc-LM5
KYSE410-I6
miR-449a mimic (nM) 0 25 50 0 25 50
MEST
Actin
34 kDa
42 kDa
KYSE150-Luc
KYSE410
Anti-miR-449a inhibitor (nM) 0 25 50 0 25 50
MEST
Actin
34 kDa
42 kDa
b
KYSE150-Luc
KYSE410
Zip-CON
Zip-449a
250 μm
Relative cell invasion (fold change)
Zip-CON
Zip-449a
***
**
c
KYSE150-Luc
Zip-CON
Zip-449a
Bioluminescence (x10^5 photons/s/cm^2/sr)
**
d
KYSE150-Luc-LM5
KYSE410-I6
KYSE150-Luc
KYSE410
miR-CON
miR-449a
Zip-CON
Zip-449a
E-cadherin
Vimentin
Snail
p-ERK (T202/Y204)
ERK
SCRIN1
RASAL1
Actin
135 kDa
57 kDa
29 kDa
44 kDa
42 kDa
140 kDa
90 kDa
42 kDa
e
KYSE150-Luc-LM5
KYSE410-I6
KYSE150-Luc
KYSE410
miR-CON + CON
miR-449a + CON
miR-449a + MEST
Zip-CON + shCON
Zip-449a + shCON
miR-449a +shMEST
MEST
E-cadherin
Vimentin
Snail
Actin
35 kDa
135 kDa
57 kDa
29 kDa
42 kDa
f
Relative luciferase activity (% of control)
miR-CON
miR-449a (0.25 μg)
miR-449a (0.5 μg)
*
***

Figure S7

Supplementary Fig. 7. (a) ESCC cells were transiently transfected with the miR-449a mimic or inhibitor, and Western blotting was used to detect MEST expression. **(b)** Knockdown of miR-449a promotes the invasion of ESCC cells. **(c)** Bioluminescence imaging showing the effect of miR-449a knockdown on tumor metastasis (n=6). **(d)** Western blot showing the expression of EMT markers and p-ERK when the expression of miR-449a was manipulated. **(e)** MEST mediated the effect of miR-449a on the expression of EMT markers. **(f)** Luciferase activity in ESCC cells transfected with increasing doses of miR-449a-expressing plasmids. Bars, SD; *, $P < 0.05$; **, $P < 0.01$; ***, $P < 0.001$, the student's *t* test.

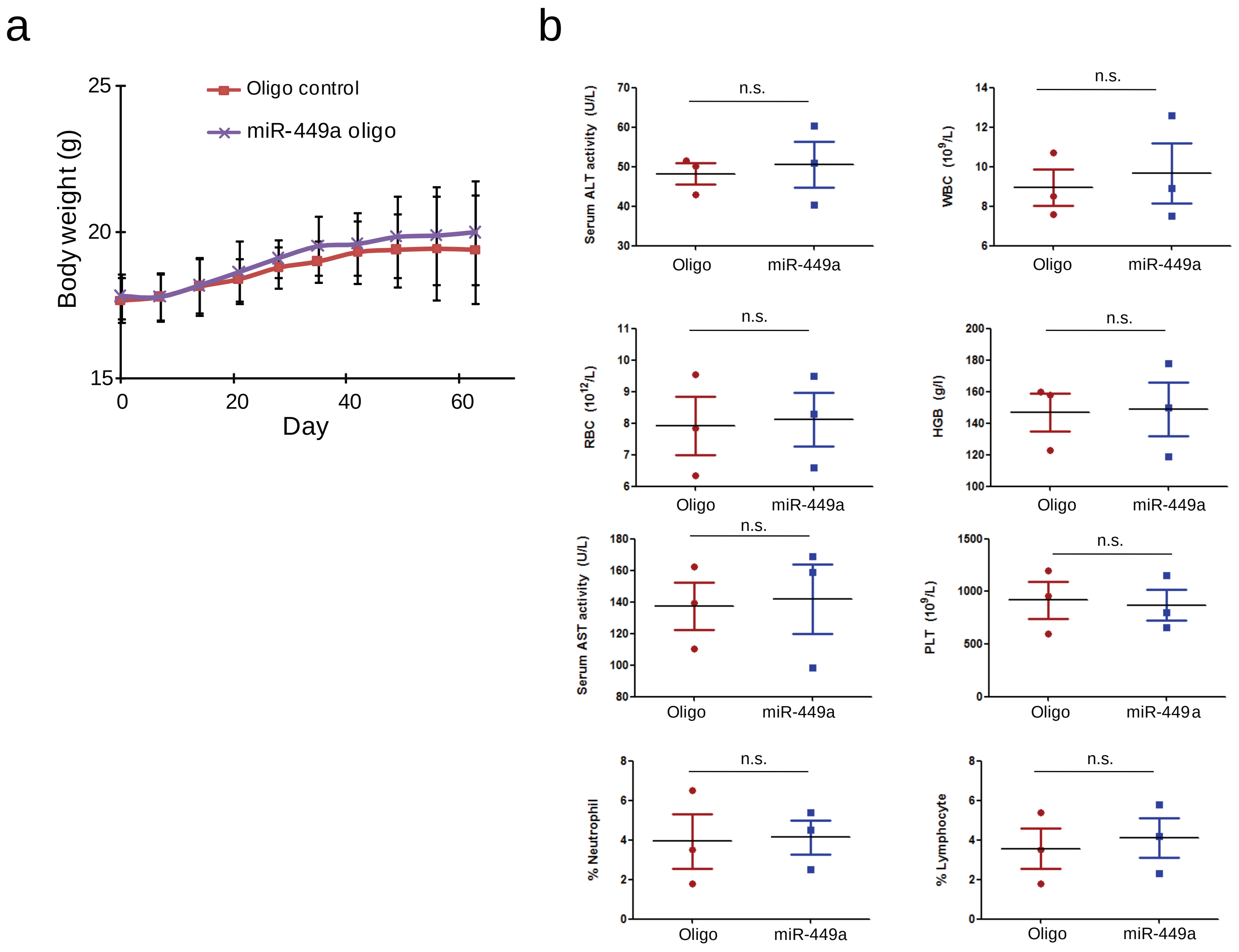

a
Oligo control
miR-449a oligo
Body weight (g)
Day
b
n.s.
Serum ALT activity (U/L)
WBC (10^9/L)
RBC (10^12/L)
HGB (g/l)
Serum AST activity (U/L)
PLT (10^9/L)
% Neutrophil
% Lymphocyte
Oligo
miR-449a

Figure S8

Supplementary Fig. 8. (a) The body weights of mice were monitored. **(b)** Blood biochemistry showing the comparison of serum ALT and AST levels between the miR-449a-treated and control groups. No significant differences were detected in terms of blood cell counts, including white blood cells (WBC), red blood cells (RBC), hemoglobin (HGB), platelets (PLT), neutrophils and lymphocytes (n=3).

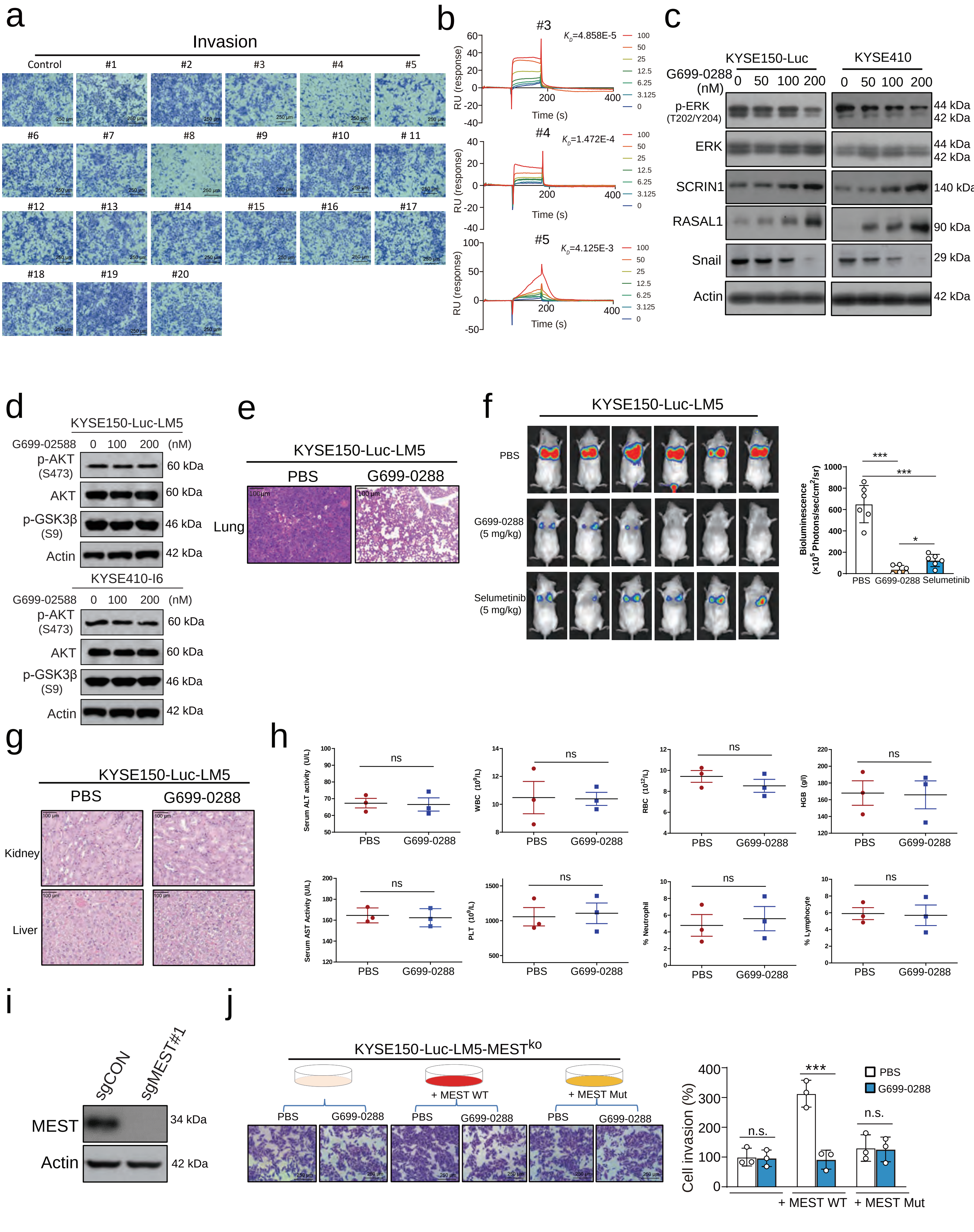

a
Invasion
Control
#1
#2
#3
#4
#5
#6
#7
#8
#9
#10
11
#12
#13
#14
#15
#16
#17
#18
#19
#20
b
#3
K_D=4.858E-5
#4
K_D=1.472E-4
#5
K_D=4.125E-3
RU (response)
Time (s)
100
50
25
12.5
6.25
3.125
0
c
KYSE150-Luc
KYSE410
G699-0288 (nM)
0 50 100 200
p-ERK (T202/Y204)
ERK
SCRIN1
RASAL1
Snail
Actin
44 kDa
42 kDa
140 kDa
90 kDa
29 kDa
d
KYSE150-Luc-LM5
G699-02588
0 100 200 (nM)
p-AKT (S473)
AKT
p-GSK3β (S9)
Actin
60 kDa
46 kDa
42 kDa
KYSE410-I6
e
KYSE150-Luc-LM5
PBS
G699-0288
Lung
f
KYSE150-Luc-LM5
PBS
G699-0288 (5 mg/kg)
Selumetinib (5 mg/kg)
Bioluminescence (×10^5 Photons/sec/cm^2/sr)
***
*
g
KYSE150-Luc-LM5
Kidney
Liver
h
Serum ALT activity (U/L)
WBC (10^9/L)
RBC (10^12/L)
HGB (g/l)
Serum AST Activity (U/L)
PLT (10^9/L)
% Neutrophil
% Lymphocyte
ns
i
sgCON
sgMEST#1
MEST
Actin
34 kDa
42 kDa
j
KYSE150-Luc-LM5-MEST^ko
+ MEST WT
+ MEST Mut
Cell invasion (%)
n.s.
***
PBS
G699-0288

Figure S9

Supplementary Fig. 9. (a) Boyden chamber assay showing the effect of the top 20 candidate compounds on ESCC cell invasion. **(b)** Representative SPR analyses indicating the binding activity of the 20 candidate compounds to the MEST protein. **(c)** Western blotting analyses of the effect of G699-0288 on the SRCIN1/RASAL1-ERK-Snail signaling pathway in ESCC cells. **(d)** Western blotting analyses showing that G699-0288 had no effect on p-AKT and p-GSK3β in ESCC cells. **(e)** Histological analysis of lungs from mice treated with G699-0288 (n=6). **(f)** Bioluminescence imaging showing the effect of G699-0288 or Selumetinib under the same dose on tumor metastasis (n=6). **(g)** The toxicity of G699-0288 was evaluated by monitoring mouse organ histology **(h)** No significant differences were detected in the blood cell counts among the groups. **(i)** Establishment of MEST-knockout cell line. **(j)** G699-0288 exerted a markedly suppressive effect on invasion ability in KYSE150-Luc-LM5-MEST-KO-WT cells, but not in KYSE150-Luc-LM5-MEST-KO or KYSE150-Luc-LM5-MEST-KO-mut cells. Bars, SD; *, $P < 0.05$; ***, $P < 0.001$, the student's *t* test.
