# Supplementary material for: Genome-wide CRISPR/Cas9 screening identifies a targetable MEST-PURA interaction in cancer metastasis
Source: eBioMedicine. 2023 May 5;92:104587. doi: 10.1016/j.ebiom.2023.104587 (PMC10192437; doi:10.1016/j.ebiom.2023.104587)

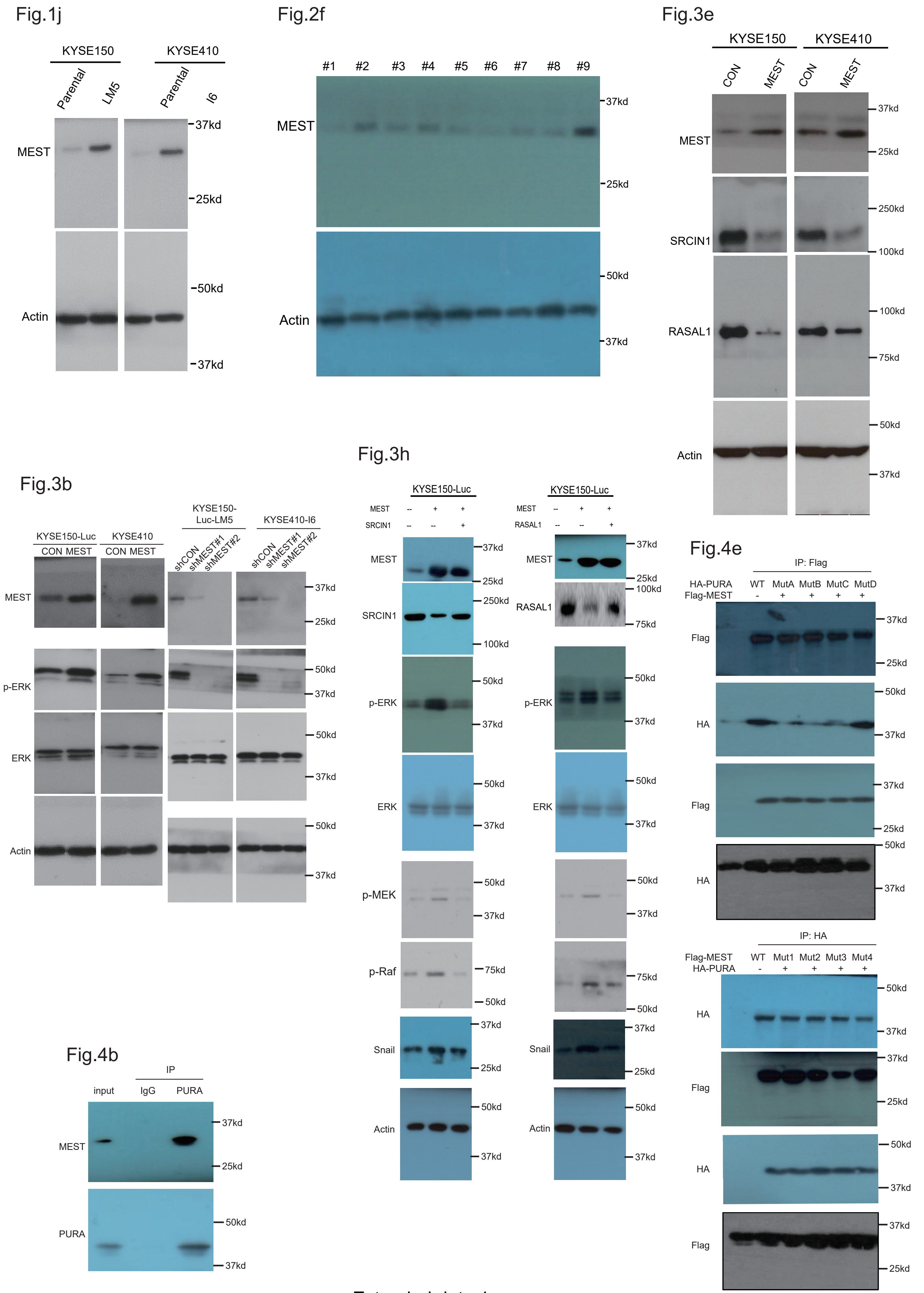

Extended data 1

Fig.4h

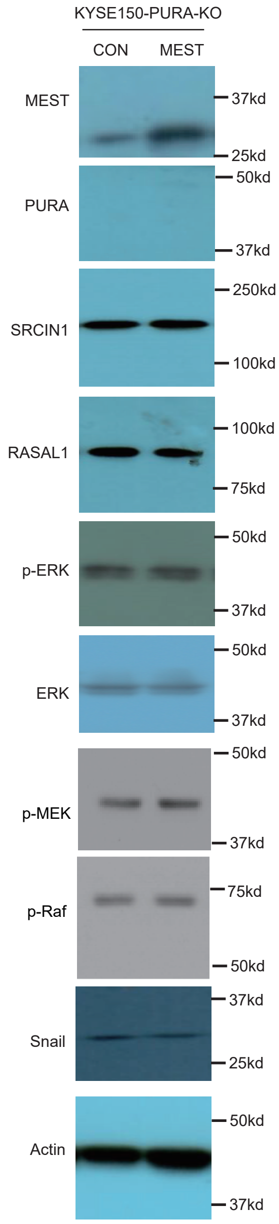

Fig.4i

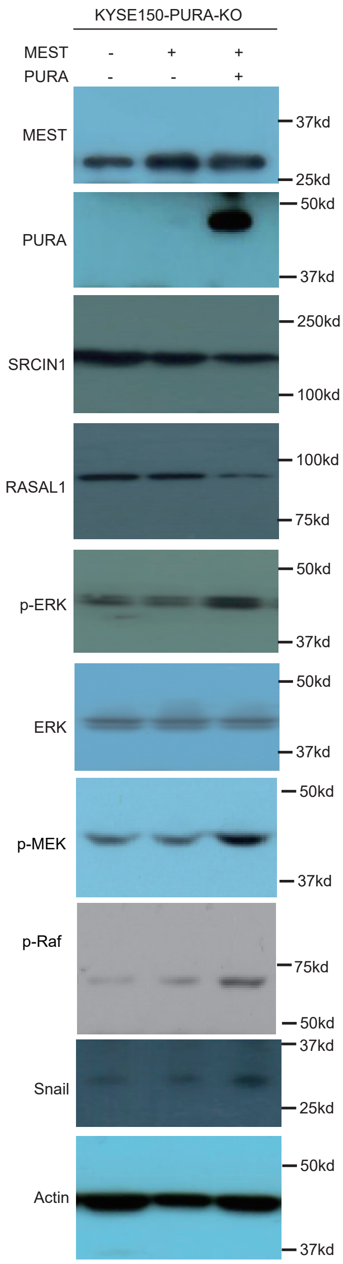

Fig.6a

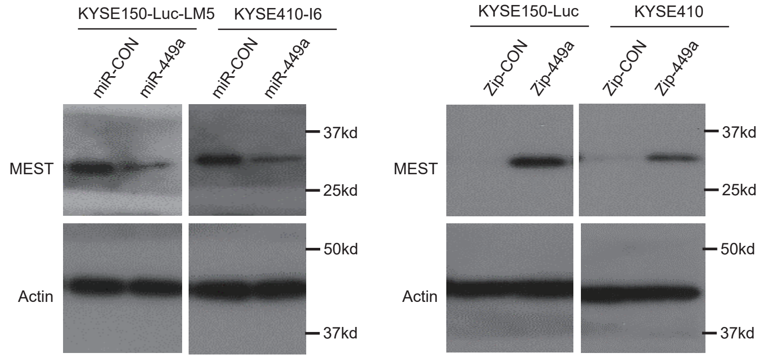

Fig.7h

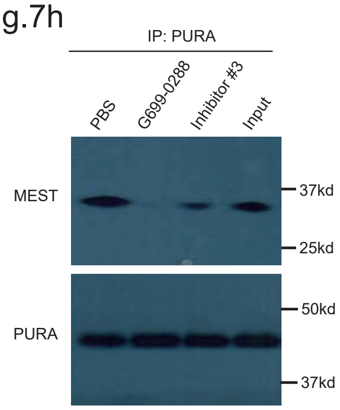

Supplementary Fig.2g

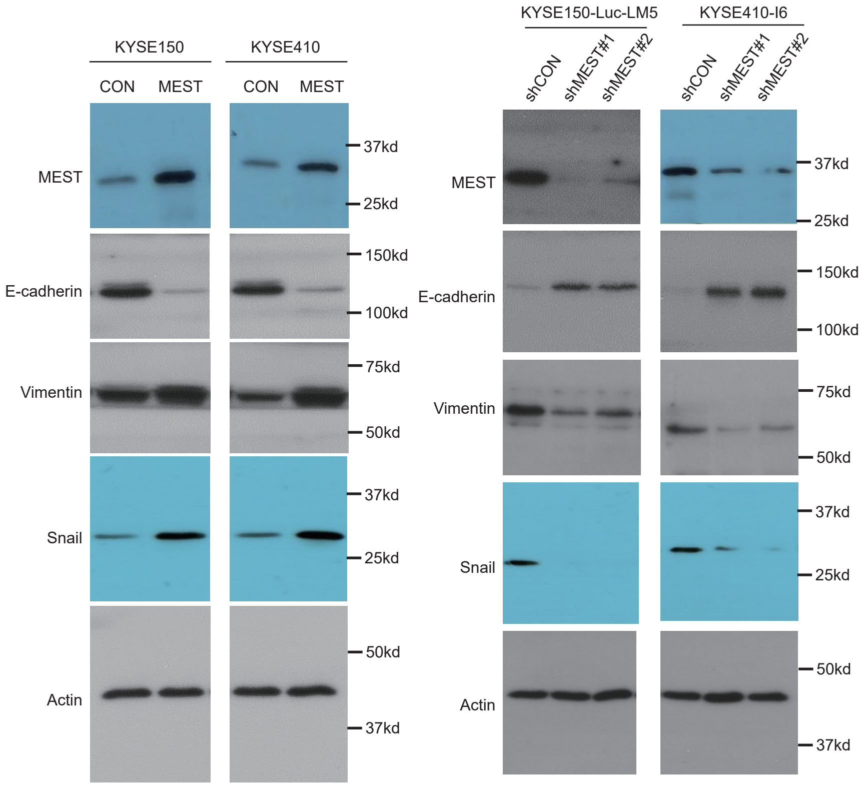

Supplementary Fig.3c

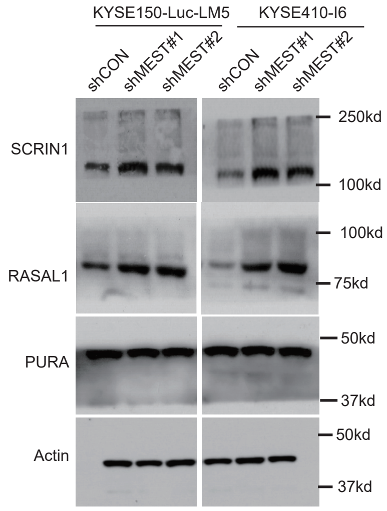

Extended data 2

Supplementary Fig.4b

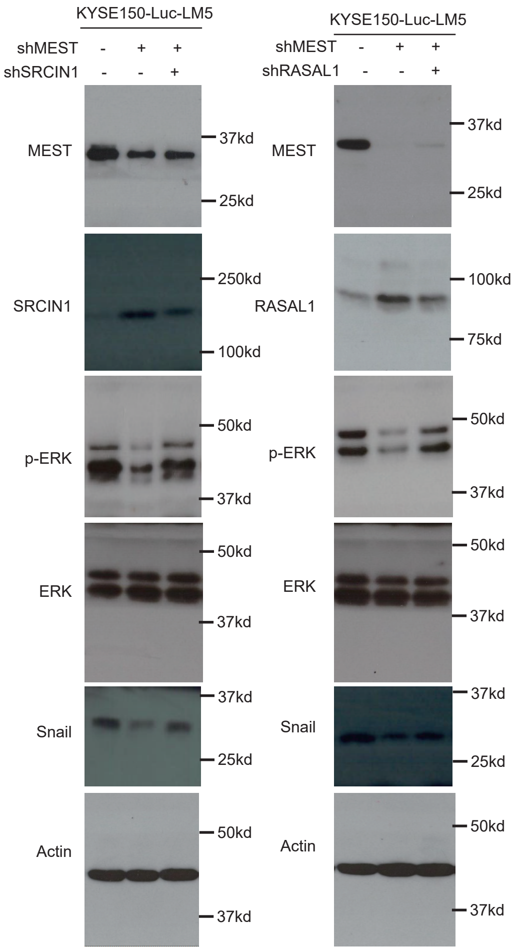

Supplementary Fig.5a

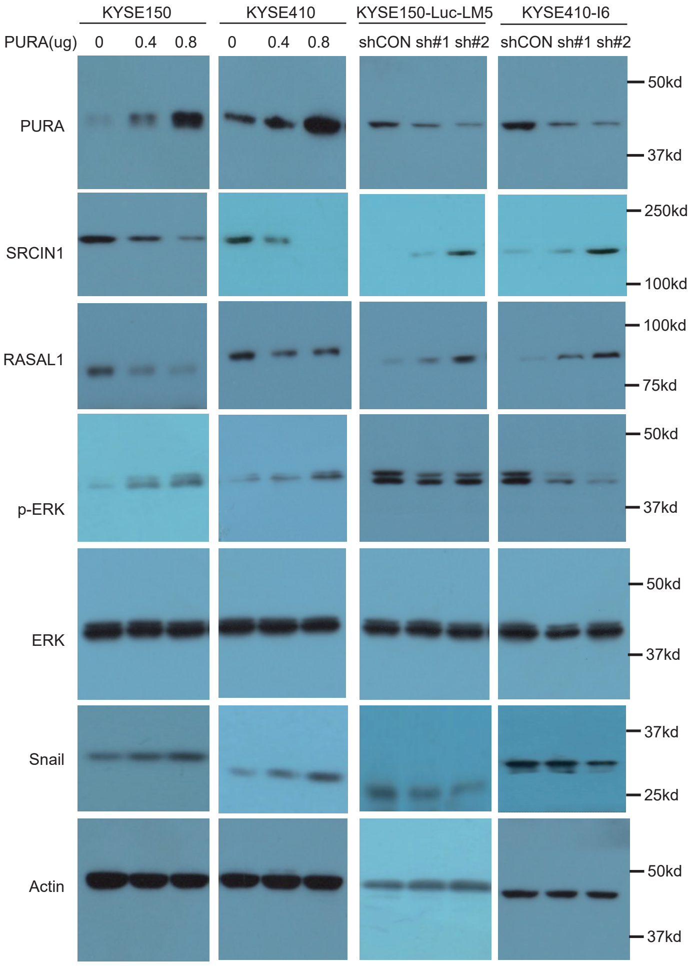

Supplementary Fig.5d

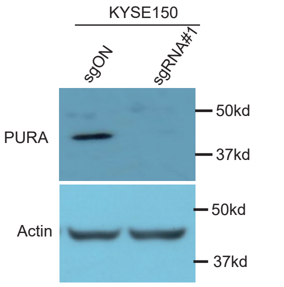

Supplementary Fig.7a

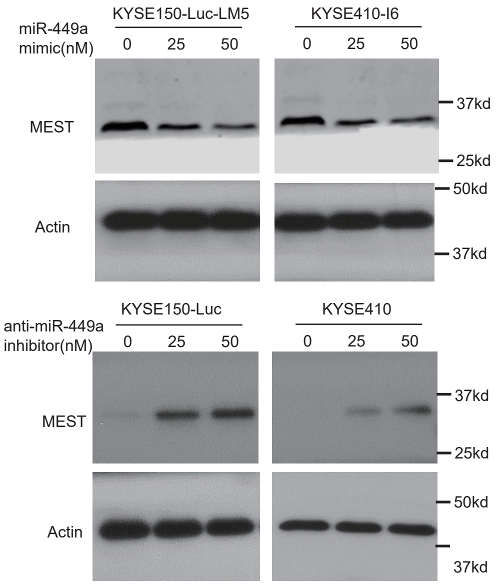

Supplementary Fig.7d

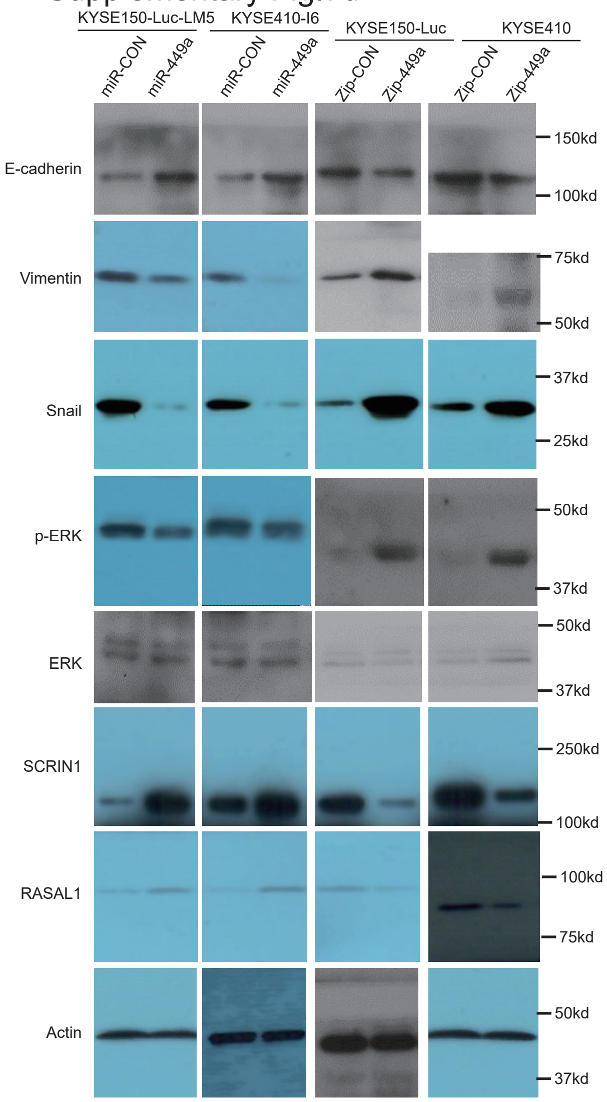

Extended data 3

Supplementary Fig.7e

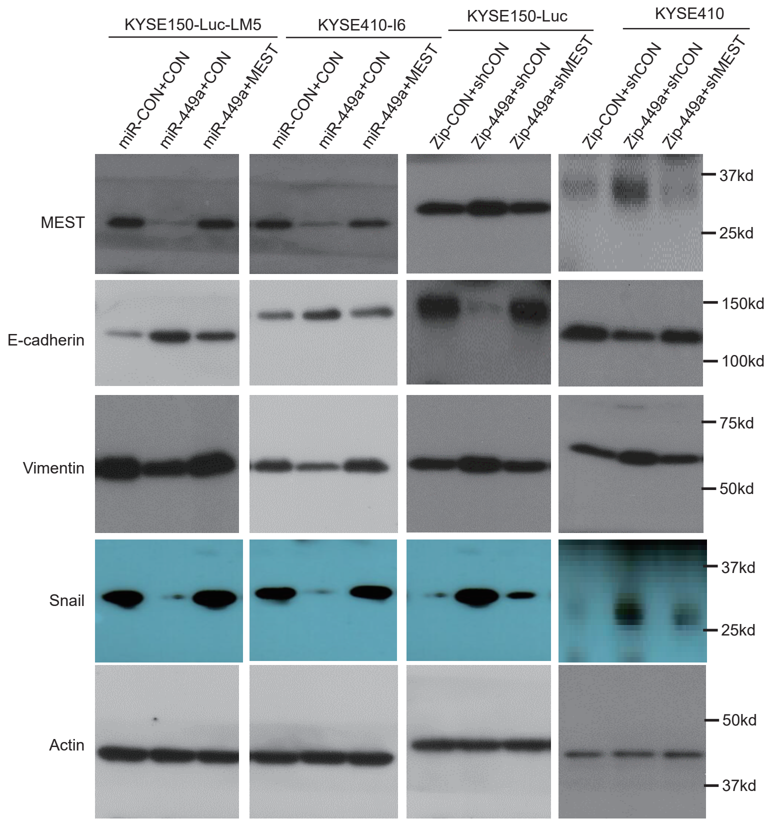

Supplementary Fig.9c

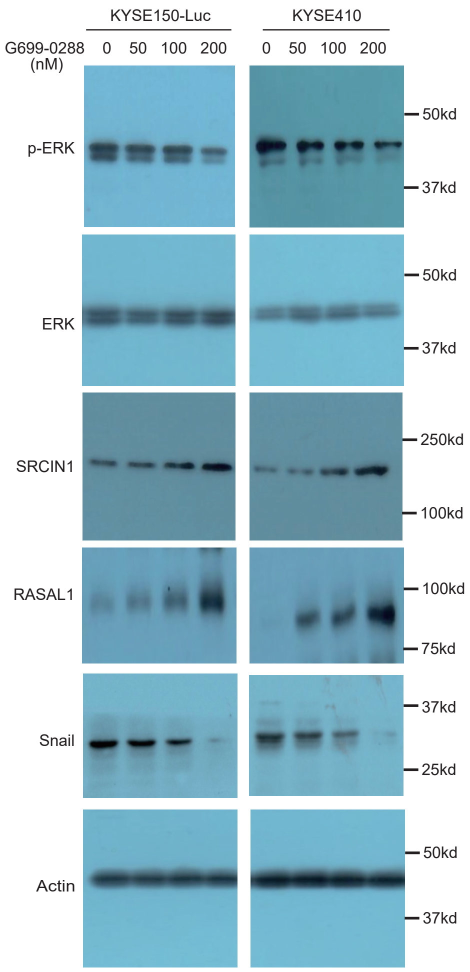

Supplementary Fig.9d

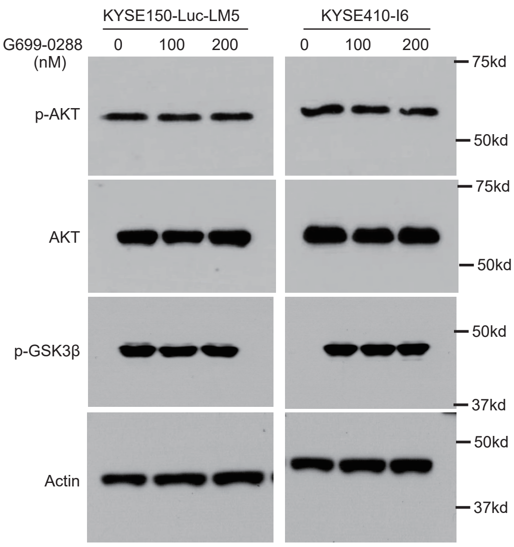

Supplementary Fig.9i

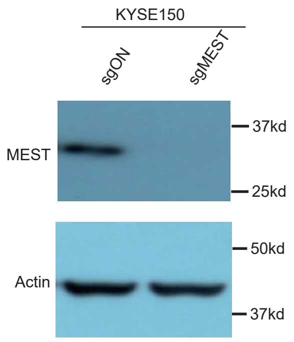

Supplement: Supplemental Western blots [file mmc15.pdf]
